# Systemic toxicity and immunotoxicity studies of glucose oxidase-loaded extracellular vesicles derived from lung cancer cells

**DOI:** 10.3389/fimmu.2026.1842887

**Published:** 2026-05-11

**Authors:** Ireneusz P. Grudzinski, Magdalena Bamburowicz-Klimkowska, Barbara Sochanowicz, Kamil Brzoska, Monika Prochorec-Sobieszek, Marzena Cabaj, Alicja Targonska, Agnieszka Stawarska, Marcin Kruszewski

**Affiliations:** 1 Department of Toxicology and Food Science, Medical University of Warsaw, Faculty of Pharmacy, Warsaw, Poland; 2 Institute of Nuclear Chemistry Technology, Centre for Radiobiology and Biological Dosimetry, Warsaw, Poland; 3 Department of Hematological Diagnostics, Pathomorphology Laboratory, Institute of Hematology and Transfusion Medicine, Warsaw, Poland; 4 Nencki Institute of Experimental Biology, Polish Academy of Sciences, Warsaw, Poland; 5 Department of Molecular Biology and Translational Research, Institute of Rural Health, Lublin, Poland

**Keywords:** extracellular vesicles, glucose oxidase, immunotoxicity, lung cancer cells, mice, systemic toxicity

## Abstract

**Introduction:**

Extracellular vesicles (EVs) are naturally occurring nanoparticles secreted by diverse cell types and are increasingly explored as delivery vehicles and immunomodulatory platforms. Rigorous preclinical safety and immunotoxicological evaluation is therefore essential prior to clinical translation. Here, using a modified OECD 423 acute toxicity class method, we assessed the toxicity of EVs loaded with glucose oxidase (GOX) along with T-dependent antibody response (TDAR) analyses in repeated-dose toxicity studies.

**Methods:**

EVs derived from human adenocarcinomic alveolar basal epithelial cells (A549) were used as enzyme carriers. In the acute toxicity study, three female BALB/c mice received a single (50 mL) intravenous (i.v.) injection of GOX-loaded EVs dispersed in phosphate-buffered saline (PBS) at a concentration of 10^8^ EVs mL^-1^. In the repeated-dose study, female BALB/c mice (n = 6 per group) were administered GOX-loaded EVs (10^4^–10^8^ EVs mL^-1^) once daily (50 mL, i.v.) over 28 days. Naïve animals treated with PBS served as vehicle controls. Additional controls included mice receiving keyhole limpet hemocyanin (KLH) as an immunostimulatory reference (a single intraperitoneal dose of 100 mg·kg^-1^ on day 14) and cyclosporine A as an immunosuppressive control (oral administration of 100 mg·kg^-1^ on days 1, 7, and 14).

**Results:**

Across all regimens, no mortality or treatment-related clinical deterioration was observed. Animals maintained normal body-weight trajectories, and functional assessments revealed no abnormalities in neuromotor performance or general behavior. Histopathological evaluation of major organs did not identify treatment-associated adverse effects. TDAR analysis demonstrated selective modulation of humoral immune responses after repeated exposure to GOX-loaded EVs. Total circulating IgM levels were significantly elevated on days 21 and 28 (approximately 1.5-fold increase; p = 0.0001) relative to controls, whereas IgG concentrations remained unchanged. GOX-loaded EV treatment induced a chemokine-biased transcriptional response, characterized by upregulation of Cxcl9 and modest increases in Cxcl3, Cxcl16, Ccl5, Ccl12, and Ccl17, while canonical pro-inflammatory cytokines showed minimal changes.

**Discussion:**

GOX-loaded EVs demonstrate a favorable short-term *in vivo* safety profile and a constrained immunological signature, engaging immune pathways in a context-dependent manner rather than acting as classical immune adjuvants, thereby supporting their potential as controllable immunomodulatory therapeutic platforms.

## Introduction

Extracellular vesicles (EVs) are naturally occurring nanoparticles secreted by cells that play a key role in various immune functions, contributing to both immune activation and suppression. EVs are involved in such immune processes as activation, regulation, suppression, tolerance, inflammation, autoimmunity, and responses to infectious diseases (1). Notably, EVs facilitate communication between tumor cells and immune cells, thus promoting tumor progression and metastasis (2). Given their capacity to deliver bioactive molecules, including proteins, lipids, and nucleic acids, EVs are increasingly being explored as potential therapeutic agents (3, 4). It has to be emphasized that the origin of EVs is a critical factor that influences both their biological effects and toxicity profile. EVs can be derived from a variety of cellular sources, including mesenchymal stem cells (MSCs), immune cells, epithelial cells, and even cancer cells. Each source imparts distinct characteristics to the EVs. For instance, MSC-derived EVs are widely studied for their regenerative and anti-inflammatory properties (5, 6). As a result, careful selection of the source is crucial to minimize unwanted side effects. In addition, the methods used for EV purification and characterization are crucial to obtaining homogeneous and uncontaminated vesicles. Common isolation techniques, such as ultracentrifugation and size exclusion chromatography, yield EVs with varying levels of purity. Contaminants such as cell debris, protein aggregates, and apoptotic bodies can trigger immune responses or toxicity unrelated to the EVs themselves, thus necessitating rigorous purification procedures (7).

EVs play a crucial role in immune regulation by facilitating antigen presentation and modulating immune cell functions. For example, dendritic cell (DC)-derived EVs present antigens to T cells, initiating adaptive immune responses. These EVs carry major histocompatibility complex (MHC) molecules and co-stimulatory proteins necessary for T-cell activation (8). Additionally, EVs transport bioactive molecules, such as cytokines and microRNAs, between cells, affecting immune signaling and cellular responses (1). While many functions of lung cancer-derived EVs are immunosuppressive, certain modifications or conditions can alter their ability to activate immune responses. For instance, heat stress or genetic modifications, such as the overexpression of Rab27a or CD40L, can induce lung cancer-derived exosomes to promote maturation of dendritic cells (DCs) (9). Mature DCs, in turn, enhance antigen presentation, which leads to activation of tumor-specific cytotoxic T lymphocytes. This process involves the transfer of tumor antigens via exosomes to DCs, which present these antigens to T cells, eliciting an immune response against the tumor (9, 10). Lung cancer-derived EVs typically exhibit immunosuppressive functions that support tumor progression. These EVs suppress immune responses by impairing the function of dendritic cells, T cells, natural killer (NK) cells, and macrophages, while also promoting the expansion of immunosuppressive cell populations, such as regulatory T cells (Tregs) and myeloid-derived suppressor cells (MDSCs) (11, 12). For instance, exosomes derived from Lewis lung carcinoma (LLC) cells have been shown to block the differentiation of myeloid precursors into functional DCs, thereby reducing the expression of maturation markers such as CD80, CD86, and MHC-II on DCs. This impairs the antigen-presenting capacity of DCs and leads to T-cell anergy, contributing to immune evasion and tumor progression (10). Furthermore, lung cancer-derived exosomes can express programmed death-ligand 1 (PD-L1), which interacts with PD-1 receptors on T cells, inhibiting their cytokine production and triggering apoptosis in activated T cells. This mechanism further helps tumor cells evade immune surveillance and diminish the effectiveness of cytotoxic T lymphocytes (2, 13). Furthermore, exosomes from lung cancer cells can promote the differentiation of CD4^+^ T cells into FOXP3^+^ regulatory T cells (Tregs) and activate MDSCs, both of which contribute to an immunosuppressive tumor microenvironment that hinders effective antitumor immunity (10, 14). This process further reduces the population of cytotoxic T cells capable of targeting tumor cells, contributing to immune evasion (15). Additionally, tumor-derived exosomes can induce apoptosis in activated CD8^+^ effector T cells through the Fas/Fas ligand pathway. By expressing Fas ligand (FasL) on their surface, these exosomes interact with the Fas receptor on T cells, triggering apoptotic signaling cascades (11, 12).

In the context of inflammation and autoimmune diseases, EVs play a key role in modulating inflammatory responses and pathogenesis. In conditions such as rheumatoid arthritis and lupus, EVs can carry pro-inflammatory cytokines and autoantigens, exacerbating chronic inflammation and tissue damage. Conversely, MSC-derived EVs have shown potential anti-inflammatory properties, making them promising candidates for treating inflammatory conditions and promoting tissue repair. In the tumor microenvironment, lung cancer-derived EVs contribute to inflammation by transferring pro-inflammatory cytokines, chemokines, and other signaling molecules, thereby amplifying inflammatory signaling pathways that promote tumor growth. This chronic inflammation disrupts tissue homeostasis and supports tumor progression. For example, EVs can carry proteins like epidermal growth factor receptor (EGFR), which has been implicated in distinguishing non-small cell lung cancer (NSCLC) from chronic lung inflammation (11, 16). The role of EVs in modulating immune tolerance and their potential contribution to autoimmune diseases is complex and still not fully understood. While direct evidence linking EVs to autoimmune disease initiation is limited, their ability to influence immune tolerance suggests that they may have an impact on the occurrence of autoimmune-like responses. By altering the function of antigen-presenting cells and promoting the expansion of Tregs, EVs may create an immunosuppressive environment that disrupts normal immune surveillance and immune homeostasis, which could potentially contribute to the development of autoimmune disorders. However, more research is needed to fully elucidate these mechanisms (13, 16).

The unique properties of EVs have led to growing interest in their potential therapeutic applications, particularly in cancer immunotherapy. Engineered EVs offer a promising platform for targeted drug delivery, enabling the direct transport of immunomodulatory agents to specific cells or tissues (17). Additionally, EVs are being explored as vaccines to present tumor-associated antigens, potentially eliciting robust immune responses against cancer cells or pathogens (18). Several clinical trials are currently investigating the efficacy of EV-based therapies in treating cancer and autoimmune diseases, highlighting their potential as innovative therapeutic tools (1). A notable therapeutic agent under multiple laboratory investigations is glucose oxidase (GOX), an enzyme that catalyzes the oxidation of glucose to gluconic acid and hydrogen peroxide (H_2_O_2_), selectively targeting cancer cells due to their increased glucose metabolism (19–21). By depleting glucose levels within the tumor microenvironment and generating reactive oxygen species (ROS), GOX induces oxidative stress in cancer cells that damages DNA, proteins, and lipids, leading to cell death (22, 23). The generated H_2_O_2_ can also potentiate the effects of chemotherapy, creating a more acidic environment and sensitizing tumor cells to chemotherapeutic agents (24, 25). Owing to these synergistic and multimodal anticancer effects, GOX has emerged as a promising component of innovative cancer treatment strategies.

Lung cancer constitutes one of the leading oncological causes of morbidity and mortality worldwide, with a persistently high fatality rate predominantly reflecting late-stage detection, aggressive tumor biology, and the limited durability of currently available therapeutic interventions (26, 27). Despite notable advances across contemporary oncological treatment approaches, clinical outcomes remain suboptimal for a substantial proportion of patients treated with current standard-of-care modalities, including chemotherapy, immunotherapy and radiotherapy, largely due to therapy resistance, pronounced intratumoral heterogeneity and treatment-associated systemic toxicity (28, 29). Collectively, these limitations underscore the urgent need for development of novel, biologically informed therapeutic approaches capable of improving antitumor efficacy while minimizing systemic toxicity. In this context, glucose oxidase (GOX)-loaded extracellular vesicles (EVs) have emerged as promising drug delivery platforms; however, their clinical translation - particularly when derived from malignant cells - raises significant safety concerns. Lung cancer-derived EVs are known to carry oncogenic proteins, nucleic acids, and immunoregulatory factors that may affect not only tumor progression but also systemic immune homeostasis (30, 31). Accumulating evidence also indicates that tumor-derived EVs can induce immunosuppressive effects, promote chronic inflammation or dysregulate immune cell function, thereby posing a risk of unintended immunotoxicity following systemic administration (32, 33). Furthermore, EV biodistribution and accumulation in off-target organs may result in systemic toxicity, which remains insufficiently characterized in current preclinical studies (34). Therefore, before EV-based medicinal products incorporating GOX or other bioactive cargos can be considered for clinical application, a comprehensive preclinical toxicological evaluation is essential. In particular, a thorough assessment of systemic toxicity and immunotoxicity is required to establish a robust safety profile and to support the rational development of EV-based therapeutics for cancer treatment, including applications in drug delivery, immunotherapy, and regenerative medicine (35, 36).

Here, we report comprehensive systemic toxicity and immunotoxicity studies of GOX-loaded EVs derived from A549 lung cancer cells. These studies were conducted in accordance with OECD Test Guideline 407 and were informed by the regulatory principles underlying the T-cell-dependent antibody response (TDAR) model. In addition, the study design adhered to the recommendations of the ICH S8 Guideline, which defines the requirements for the evaluation of immunotoxic potential of novel medicinal products. As part of these investigations, extensive histopathological documentation was generated, providing detailed insights into the preclinical safety profile of the investigated GOX-loaded EVs.

## Materials and methods

### Isolation and characterization of extracellular vesicles

Human adenocarcinomic alveolar basal epithelial cell line - A549 (ATCC CCL-185) was obtained from the American Type Culture Collection (ATCC, Manassas, VA, USA). The A549 cells were grown as an adherent monolayer in F-12K medium (Kaighn’s modification of Ham’s F-12 medium; Gibco, Paisley, supplemented with 10% fetal bovine serum (FBS; Gibco, Paisley, UK) and antibiotics (streptomycin, 50 µg·mL^-1^; amphotericin B, 1.25 µg·mL^-1^; gentamicin, 50 µg·mL^-1^; penicillin, 50 µg·mL^-1^) (Gibco, Paisley, UK). Prior to EVs isolation, the standard media was replaced with a 10% exosome-depleted FBS media (One ShotTM format, Gibco, Paisley, UK), and A549 cells were incubated for a further 3 days in T225 culturing flasks. The cell culture media was harvested from the A549 cells and isolation of extracellular vesicles was performed using gradient centrifugation and ultracentrifugation methods, as described in detail elsewhere (37). The as-collected EVs were characterized separately based on the MISEV2018 guidelines 7 (37, 38). Protein concentration was determined using Pierce BCA Protein Assay Kit (Thermo Fisher Scientific™, United States), and following the manufacturer’s instructions. Briefly, 20 μL of each standard and sample were added to a 96-well plate. Then, 200 μL of working reagent (mixed 50 parts of BCA Reagent A with 1 part of BCA Reagent B) was added to each well. The plate was covered and incubated at 37°C for 30 min. The absorbance was measured at 562 nm on a spectrophotometric multi-well plate reader.

Particle size and distribution analysis of extracellular vesicles were performed using nanoparticle tracking analysis (NTA) with NanoSight NS300 (Malvern Panalytical Ltd, UK) equipped with a 488nm blue laser (37). Specific biomarkers of extracellular vesicles, such as CD9, CD63 and CD81, were assayed using surface plasmon resonance (SPR) as developed in-house and described previously (39). Please note that this method was validated against Western Blot assay as routinely used for EV characteristics in our laboratories (37).

### Glucose oxidase loading into extracellular vesicles

Extracellular vesicles derived from A549 cells were suspended in phosphate-buffered saline (PBS, pH 7.2) to a protein concentration of 0.25 mg mL^-1^. The GOX enzyme was dissolved in PBS to a concentration of 0.5 mg mL^-1^. A 250 µL aliquot of the exosome suspension was measured, and 170 µL of the GOX solution was added. The mixture was subjected to optimized electroporation parameters (voltage – 100 V, pulse duration – 10 ms, number of pulses – 1, pulse intervals – no, resistance – 20 Ω) using a BTX ECM 830 electroporator (Harvard Apparatus/Harvard Bioscience, Holliston, MA, USA) and then incubated for 18 hours at room temperature to facilitate cellular uptake of GOX. The samples were centrifuged at 100,000 g for 90 minutes at 4 °C to pellet the cells, and the supernatant was discarded. The cell pellet was resuspended in 200 µL of PBS. A 50 µL aliquot of the cell suspension was used for the BCA protein assay to determine total protein content. A 100 µL aliquot of the cell suspension was analyzed by high-performance liquid chromatography (HPLC) to quantify the amount of flavin adenine dinucleotide (FAD), a cofactor essential for GOX activity. The FAD determination method has been described in detail elsewhere (20). A 50 µL aliquot of the cell suspension was subjected to nanoparticle tracking analysis to determine the size distribution and concentration of extracellular vesicles including pristine EVs and GOX-loaded EVs. More detailed characteristics of A549-derived EVs loaded with GOX were recently published (21).

### Ethics approval and consent to participate

All procedures were conducted in accordance with Directive 2010/63/EU on the protection of animals used for scientific purposes and the ARRIVE Guidelines 2.0 and were approved by the Institutional Animal Ethics Committee of the Warsaw University of Life Sciences (approval no. WAW2/077/2022).

### Acute toxicity studies

Acute toxicity of EVs loaded with glucose oxidase was tested according to the modified OECD 423 acute toxicity class method (40). In the study, three female BALB/c mice (8 weeks old, ca. 24 g body weight) received a single intravenous (i.v.) injection of GOX-loaded EVs dispersed in phosphate-buffered saline (PBS) at a dose of 50 µL (10^8^ EVs mL^-1^), as determined based on prior *in vitro* cytotoxicity and genotoxicity studies (21, 41). Mice injected (i.v.) with PBS alone served as the control group. Death, a moribund state, or a significant decline in animal well-being constituted criteria for study termination. Conversely, animal survival was used as the basis for proceeding to the next higher predefined dose level. The modified OECD 423 protocol allows for the determination of the median lethal dose (LD_50_) using 3 (minimum) to 12 (maximum) animals, according to the classification scheme. Prior to study termination, blood and tissue samples were collected for hematological, biochemical, and histopathological analyses.

### Repeated dose toxicity and immunotoxicity studies

Repeated-dose (28-day) toxicity studies assess health risks associated with short-term repeated exposure, in accordance with the ICH M3 guideline (42) on study duration prior to first-in-human trials. The modified OECD 407 protocol (43) allows for simultaneous evaluation of systemic toxicity and immunotoxicity. Immunotoxic effects of GOX-loaded EVs were assessed using endpoints derived from the T-cell-Dependent Antibody Response (TDAR) model (44, 45) in line with the ICH S8 guideline (43). The TDAR assay measured primary IgM and IgG responses to keyhole limpet hemocyanin (KLH). Total IgM, KLH-specific IgM and IgG, and KLH-specific IgG levels were determined by ELISA according to the manufacturer’s instructions (Abnova Manufacturing, Taipei City, Taiwan). In the repeated-dose toxicity and immunotoxicity study, female BALB/c mice (6–8 weeks old, 18–22 g body weight) were used. The animals were randomly assigned to 10 groups of six individuals and acclimatized for two weeks prior to the experiments (see **Supplementary Figure S1** for the experimental setup). Three dose levels of GOX-loaded EVs were evaluated, along with appropriate controls, including the vehicle (PBS), an immunostimulant (keyhole limpet hemocyanin, KLH), and an immunosuppressant (cyclosporine A). Clinical observation of the mice was performed once a week. Completion of the experiment was preceded by the collection of blood and tissue samples from the animals to perform biochemical, immunologic and histopathological analyses. The experimental groups were defined as follows:

Group 1: vehicle: PBS administered i.v. once daily (50 µL) for 28 consecutive days.

Group 2: immunostimulation: PBS administered i.v. once daily (50 µL) for 28 consecutive days; KLH administered as a single i.p. dose (50 µL; 100 mg/kg body weight) on day 14.

Group 3: immunosuppression: PBS administered i.v. once daily (50 µL) for 28 consecutive days; cyclosporine A administered orally (per os) at 100 mg/kg body weight on days 1, 7, and 14.

Group 4: immunosuppression plus immunostimulation: PBS administered i.v. once daily (50 µL) for 28 consecutive days; cyclosporine A administered orally (per os) at 100 mg/kg body weight on days 1, 7, and 14; KLH administered as a single i.p. dose (50 µL; 100 mg/kg body weight) on day 14.

Group 5: GOX-loaded EVs, dose 1 (10^4^ mL^-1^): GOX-loaded EVs were administered i.v. once daily (50 μL) for 28 consecutive days.

Group 6: GOX-loaded EVs, dose 2 (10^6^ mL^-1^): GOX-loaded EVs were administered i.v. once daily (50 μL) for 28 consecutive days.

Group 7: GOX-loaded EVs, dose 3 (10^8^ mL^-1^): GOX-loaded EVs were administered i.v. once daily (50 μL) for 28 consecutive days.

Group 8: GOX-loaded EVs, dose 1 (10^4^ mL^-1^): GOX-loaded EVs were administered i.v. once daily (50 μL) for 28 consecutive days; KLH was administered as a single i.p. dose (50 µL) of 100 mg/kg body weight on day 14.

Group 9: GOX-loaded EVs, dose 2 (10^6^ mL^-1^): GOX-loaded EVs were administered i.v. once daily (50 μL) for 28 consecutive days; KLH was administered as a single i.p. dose (50 µL) of 100 mg/kg body weight on day 14.

Group 10: GOX-loaded EVs, dose 3 (10^8^ mL^-1^): GOX-loaded EVs were administered i.v. once daily (50 μL) for 28 consecutive days; KLH was administered as a single i.p. dose (50 µL) of 100 mg/kg body weight on day 14.

### Transcriptional expression of cytokines and chemokines

Total RNA was extracted from mouse blood using the Mouse RiboPure Blood RNA Isolation Kit (ThermoFisher Scientific) according to the manufacturer’s protocol. RNA concentration was measured using Quantus Fluorometer (Promega) and QuantiFluor RNA System (Promega). The total RNA (600 ng) was reverse transcribed using iScript Advanced cDNA Synthesis Kit (BioRad) following the manufacturer’s instructions. The cDNA was diluted five times with deionized, RNAse-free H_2_O and used for expression profiling using the Cytokines and Chemokines M96 PrimePCR PCR Array (BioRad) according to the manufacturer’s instructions. Briefly: a total volume of 20 μL of PCR reaction mixture, which included 10 μL of SsoAdvanced Universal SYBR Green Supermix from BioRad, 9 μL of RNAse-free H_2_O and 1 μL of diluted template cDNA was used for each primer set in each well of the PCR array. PCR amplification was carried out using CFX96 Touch Real-Time PCR Detection System (BioRad) with an initial 2 min step at 95 °C followed by 40 cycles of 95 °C for 5 s and 60 °C for 30 s. Relative gene expression was calculated using the ΔΔCt method. Hprt, Hsp90ab1 and Tbp were used as reference controls. Calculations were done using CFX Maestro 2.3 Software (BioRad). Gene expression is presented as the change in mRNA levels relative to the control group (group 1 - PBS-treated mice). In some cases, the expression of a given cytokine in all mice tested was too low to be measured (no amplification). In such cases, expression values are missing.

### Histopathology

The collected tissues (heart, lung, liver, kidney and spleen) were fixed in 10% neutral-buffered formalin. The tissue samples were dehydrated in increasing concentrations of alcohol and acetone and embedded in paraffin. The paraffin blocks were cut into 4µm thick sections and placed on glass slides. The slides were incubated at 60 °C in a laboratory incubator for one hour, then deparaffinized and hydrated. Tissues were routinely stained with hematoxylin and eosin (H&E). Harri’s Hematoxylin stain and eosin solution were used according to the following procedure: 1. Nuclear Staining: staining in hematoxylin for 3–5 minutes. 2. Washing in running tap water until sections “blue” for 5 minutes or less. 3. Differentiation: selective removal of excess dye from the section. Dip in 1% acid alcohol (1% HCl in 70% alcohol) for a few seconds. 4. Blueing: Rinse in running tap water. Dip in ammonia water until the sections become blue, followed by a tap water wash. 5. Counterstain: Staining in 1% Eosin Y for 10 minutes. 6. Washing in tap water for 1–5 minutes. 7. Dehydration: Dehydrating in increasing concentration of alcohols. 8. Clearing: Putting slides in two xylene baths for clearing. 9. Mounting in DPX. A semi-quantitative method for evaluation of intensity chronic inflammatory infiltrates in solid organs and lymphoid hyperplasia in the spleen was applied (0 – none; 0–1 – minimal; 1 – mild; 2 – moderate. All photographs from slides with H&E were taken using the microscope camera DP72 Olympus BX63 (Olympus, Japan). Samples were reviewed by a certified pathologist. The evaluation was performed in a blinded manner.

### Statistical analysis

Statistical analysis was performed for body weight, TDAR, and cytokine data using Statistica 13.3. For the controls and treated groups, the homogeneity of variance was determined by F Welch test. Groups were compared by one-way analysis of variance (ANOVA). If statistical significance was noted, a RIR Tukey test was used for multiple comparisons of groups. Body weight in the acute toxicity study was analyzed by the t-Student test. p<0.05, differences between the groups were considered significant. Hierarchical cluster analysis was made for cytokine analysis.

## Results

### Single and repeated dose toxicity studies

**Figure 1:**
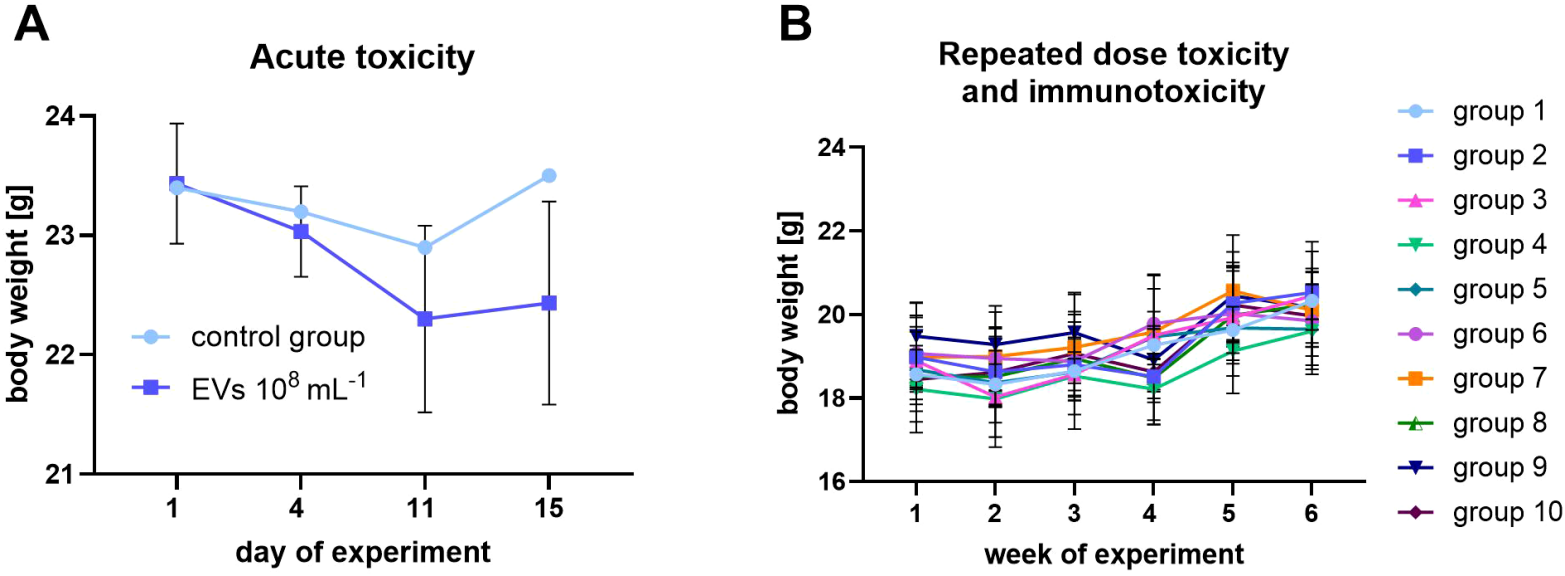
Body weight changes of BALB/c mice during acute and repeated-dose toxicity studies of A549-derived extracellular vesicles (EVs) loaded with glucose oxidase (GOX). In the acute toxicity study **(A)**, mice received a single administration of GOX-loaded EVs at a concentration of 10^8^ mL^-1^ or vehicle (PBS) control, and body weight was monitored over 15 days. This dose was selected as a biologically relevant worst-case exposure to assess acute safety rather than dose-response effects. In the repeated-dose toxicity and immunotoxicity study **(B)**, mice were treated according to the experimental design described in the Materials and Methods, comprising ten groups (groups 1–10) differing in EV doses, treatment schedule, and respective controls. Body weight was recorded weekly for six weeks. Data are presented as mean ± SD. Statistical analysis revealed no significant differences in body weight between control (group 1) and GOX-loaded EV-treated groups at any time point.

The single-dose toxicity study established the acute safety profile of the GOX-loaded EVs and enabled estimation of the median lethal dose (LD_50_) range, thereby informing dose selection for the subsequent 28-day repeated-dose regimen. Data generated in the acute test directly shaped the dosing strategy applied in the longer-term study. Across both the acute and repeated-dose experiments, mouse body weight was monitored longitudinally. As shown in **Figure 1**, animals receiving A549-derived GOX-loaded EVs exhibited body-weight trajectories indistinguishable from those of control cohorts in both the single-dose and 28-day settings, indicating an absence of overt systemic toxicity under the evaluated conditions. In a single acute dose, a modest, transient reduction in body weight was noted during the 14-day period following a single intravenous administration of GOX-derived EVs (10^8^ mL^-1^). Importantly, no deviations in spontaneous behavior, activity levels, posture, grooming, social interactions, or exploratory patterns were observed in mice. Feed and water consumption remained within expected physiological ranges, with no signs of anorexia, dehydration, distress-associated behaviors, or circadian disruption. No mortality, pre-terminal states, or clinical abnormalities requiring early intervention occurred. Because this dose induced only a mild and non-progressive physiological effect, it was selected as the upper dose for the OECD-compliant 28-day repeated-dose toxicity study.

In the repeated-dose setting, mice receiving GOX-loaded EVs displayed stable body-weight trajectories and preserved normal behavioral repertoires throughout the 28-day observation period. No indicators of neurological dysfunction (e.g., impaired coordination, tremors, altered gait), autonomic abnormalities (e.g., piloerection, respiratory distress), or changes in reactivity to external stimuli were detected. Feed and water intake remained comparable to that of controls, supporting preserved systemic metabolic homeostasis. Neither EV-treated animals nor the respective clinical abnormalities, including dermatological lesions, injection-site reactions, gastrointestinal disturbances, or signs of systemic inflammation, were noted. Throughout the entire study, no mortality, pre-terminal conditions, or adverse clinical manifestations were recorded. Collectively, these data support a favorable tolerability profile of GOX-loaded EVs across both acute and repeated-dose exposure paradigms, with no evidence of behavioral, clinical, or systemic toxicity under the tested conditions.

### Histopathological analysis

**Figure 2:**
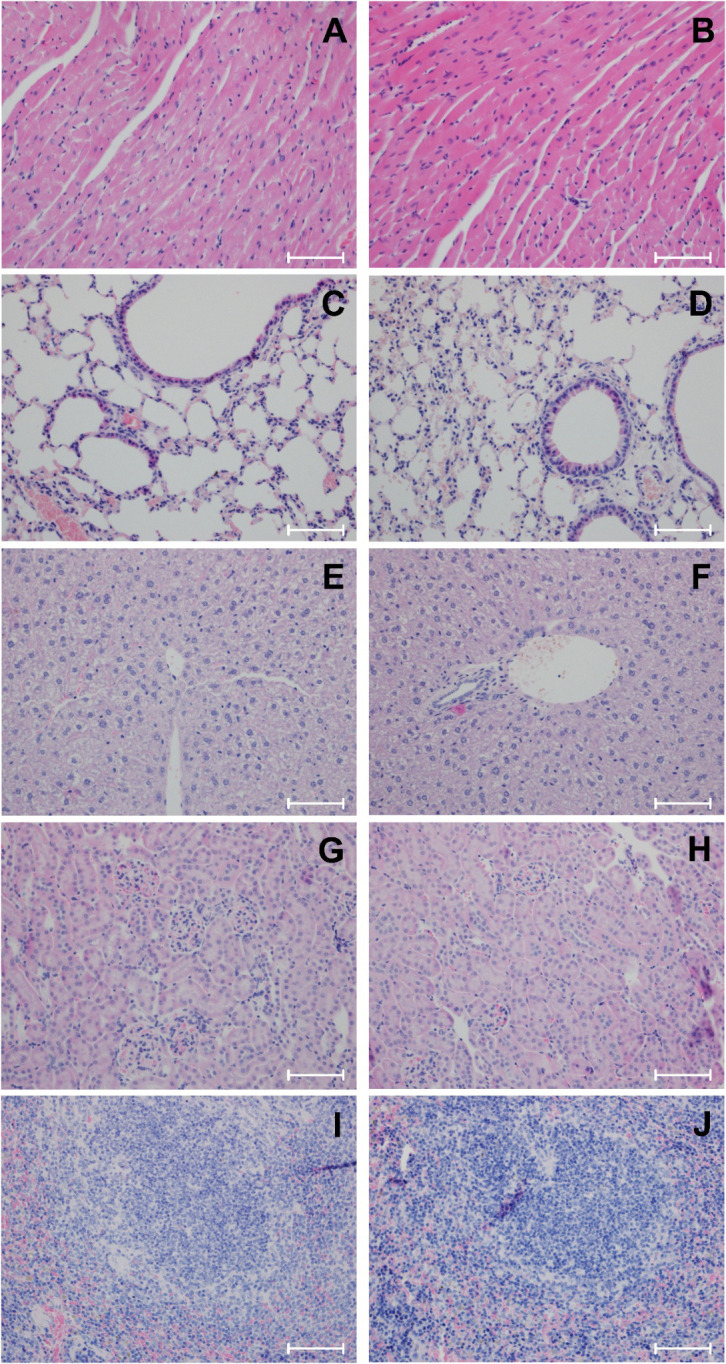
Representative resection specimens of BALB/c mice treated with A549-derived EVs loaded with glucose oxidase based on the acute toxicity protocol (H&E staining, x200, scale bar = 50 μm). PBS-treated control mice **(A, C, E, G, I)** and GOX-loaded EV-treated mice **(B, D, F, H, J)**. **(A, B)** Myocardium: preserved normal myocardial architecture with no evidence of necrosis, degenerative changes, fibrosis, hypertrophy, or inflammatory infiltrates (inflammatory infiltrates grade 0, none). **(C, D)** Lung: intact alveolar and interstitial architecture with no necrosis, degenerative changes, alveolar damage, emphysema, fibrosis, or inflammatory infiltrates (inflammatory infiltrates grade 0, none). **(E, F)** Liver: normal hepatic architecture without necrosis, degenerative changes, bile stasis, fibrosis, or inflammatory infiltrates (inflammatory infiltrates grade 0, none). **(G, H)** Kidney: preserved renal histology with normal glomerular and tubular structure; no necrosis, degenerative changes, fibrosis, or inflammatory infiltrates (inflammatory infiltrates grade 0, none). **(I, J)** Spleen: normal splenic architecture with no necrosis, degenerative changes, congestion, fibrosis, or white pulp hyperplasia (grade 0, none). Histopathological scoring: Inflammatory infiltrates: 0, none; 0–1, minimal; 1, mild; 2, moderate. White pulp hyperplasia (spleen): 0, none; 0–1, minimal; 1, low; 2, moderate.

**Figure 3:**
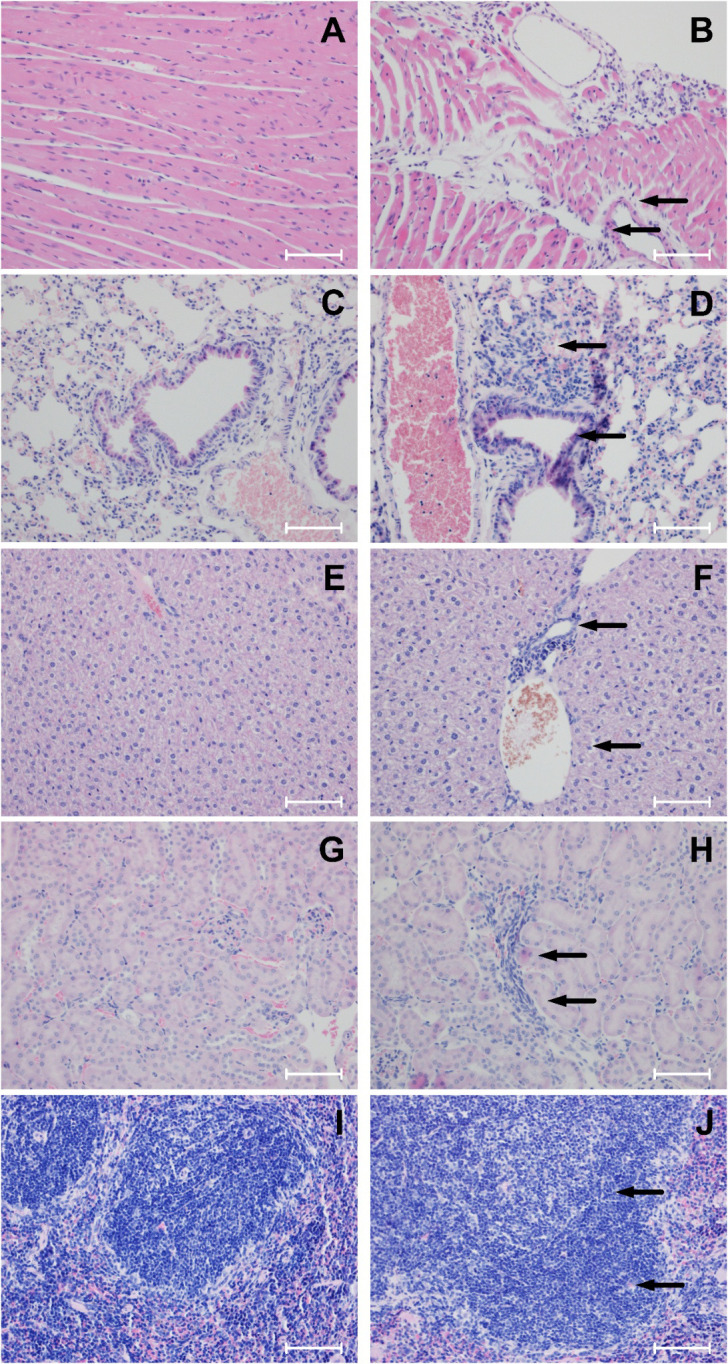
Representative resection specimen in BALB/c mice treated with A549-derived EVs loaded with glucose oxidase in repeated dose (28-days) toxicity study (H&E staining, x200, scale bar = 50 μm). PBS-treated control mice: **(A)** Myocardium: preserved normal architecture; no necrosis, degenerative changes, fibrosis, hypertrophy, or inflammatory infiltrates (inflammatory infiltrates grade 0, none). **(C)** Lung: intact alveolar and interstitial architecture; no necrosis, degenerative changes, alveolar damage, emphysema, fibrosis, or inflammatory infiltrates (grade 0, none). **(E)** Liver: normal hepatic architecture; no necrosis, degenerative changes, bile stasis, fibrosis, or inflammatory infiltrates (grade 0, none). **(G)** Kidney: preserved glomerular and tubular structure; no necrosis, degenerative changes, fibrosis, or inflammatory infiltrates (grade 0, none). **(I)** Spleen: normal architecture; no necrosis, degenerative changes, congestion, fibrosis, or white pulp hyperplasia (grade 0, none). GOX-loaded EV-treated mice: **(B)** Myocardium: preserved myocardial fibers with focal chronic inflammatory infiltrates in pericardial adipose tissue (arrows), corresponding to inflammatory infiltrates grade 1 (mild). **(D)** Lung: chronic peribronchiolar and perivascular inflammatory infiltrates (arrows) with preserved alveolar and interstitial architecture (inflammatory infiltrates grade 1, mild). **(F)** Liver: normal hepatocyte morphology with mild chronic inflammatory infiltrates in portal spaces (arrows), corresponding to inflammatory infiltrates grade 1 (mild). **(H)** Kidney: preserved glomeruli and tubules with minimal chronic interstitial inflammatory infiltrates consisting of single, scattered cells (arrows), corresponding to inflammatory infiltrates grade 0–1 (minimal). **(J)** Spleen: white pulp stimulation with enlarged lymphoid follicles and prominent germinal centers (arrows), corresponding to white pulp hyperplasia grade 2 (moderate). Histopathological scoring: Inflammatory infiltrates: 0, none; 0–1, minimal; 1, mild; 2, moderate. White pulp hyperplasia (spleen): 0, none; 0–1, minimal; 1, low; 2, moderate.

Histopathological evaluation represented a primary endpoint in both the acute and repeated-dose toxicity studies, enabling systematic assessment of potential organ-specific adverse effects associated with exposure to GOX-loaded EVs. Comprehensive analyses were performed on major mouse organs, including the heart, lungs, liver, kidneys, and spleen, collected post-mortem from all animals enrolled in the study arms. Tissue processing and H&E staining were conducted following standardized OECD-compliant procedures to ensure methodological rigor and reproducibility. Representative micrographs are provided in **Figures 2** and **3** and **Supplementary Materials** (**Supplementary Table 1**, **S2**), illustrating both organ sections devoid of pathological alterations and those containing subtle structural features flagged for closer examination. Evaluation encompassed a broad spectrum of histopathological parameters, including parenchymal integrity, evidence of degeneration or necrosis, inflammatory cell infiltration, vascular or stromal abnormalities, and architectural disruptions suggestive of organ-specific toxicity. In the acute toxicity study, no necrosis, degenerative lesions, inflammatory infiltrates, or morphological abnormalities were identified in any examined organs from either control animals or those treated with GOX-loaded EVs (**Figure 2**; **Supplementary Table 1**). Cardiac myofibers retained normal morphology without evidence of necrosis, degenerative changes, cytoplasmic vacuolization, interstitial edema. fibrosis, hypertrophy or inflammatory infiltrates. Hepatic lobular architecture remained intact, with preserved hepatocyte morphology and no signs of necrosis, degenerative changes, bile stasis, fibrosis and inflammatory infiltrates. Renal tissue showed well-preserved glomerular structures and tubular epithelium without indications of necrosis or tubular degeneration. Similarly, pulmonary tissue demonstrated normal alveolar architecture, and the spleen exhibited physiological white-pulp and red-pulp organization without hyperplasia or depletion. These findings collectively support the absence of organ-level toxicity following a single intravenous exposure to the tested EV formulation. The histopathological normalcy observed across multiple major organ systems provides an important mechanistic complement to the behavioral, clinical, and body-weight data, collectively reinforcing the favorable acute safety profile of GOX-loaded EVs.

Histopathological assessment following the 28-day repeated-dose exposure revealed a consistent and dose-dependent pattern of tissue responses across BALB/c mice administered GOX-loaded EVs derived from A549 lung cancer cells. As in the acute study, the lowest EV dose (10^4^ mL^-1^) did not induce discernible structural abnormalities in any examined organ, indicating an absence of detectable systemic or organ-specific toxicity at this exposure level (**Supplementary Table 2**). At higher doses (10^6^ mL^-1^and 10^8^ mL^-1^), histopathological alterations were primarily localized to lymphoid-associated and perivascular compartments (**Figure 3**; **Supplementary Table 2**). The most prominent finding consisted of chronic lymphocytic infiltrates within perinephric adipose tissue, characterized by small, mature lymphocytes distributed in perivascular and stromal spaces without evidence of necrosis, fibrosis, or tissue destruction. These infiltrates suggest a mild, localized immunomodulatory response rather than overt inflammatory pathology. Correspondingly, a dose-related stimulation of secondary lymphoid architecture was observed. The spleens of EV-treated animals displayed enlargement of white-pulp compartments with expansion of lymphoid nodules and the formation of well-defined germinal centers (score 2), indicative of adaptive immune activation. The presence of polarized germinal centers with preserved follicular organization suggests a physiologically regulated B-cell response rather than dysregulated lymphoproliferation. No evidence of red-pulp congestion, sinusoidal dilation, or extramedullary hematopoiesis was detected. In the lungs, A549-derived enzyme-loaded EVs did not disrupt alveolar morphology, interalveolar septa, or microvascular integrity. Airway epithelium remained intact, and no features of pneumonitis, alveolar edema, or parenchymal remodeling were noted. Mild, chronic inflammatory infiltrates were observed in peribronchiolar and perivascular regions (score 1), composed predominantly of mononuclear cells with occasional perivascular cuffing. These changes were minimal, non-progressive, and lacked associated epithelial injury, suggesting low-grade immune engagement rather than classical inflammatory lung toxicity. Importantly, throughout all dose groups, no systemic toxicity was detected in the heart or liver. Cardiac tissue exhibited preserved myofibrillar alignment, intact nuclei, and no signs of vacuolization, myocarditis, or vascular injury. Hepatic histology demonstrated normal lobular architecture with intact hepatocyte plates, absence of portal or periportal inflammation, and no evidence of microvesicular or macrovesicular steatosis, hepatocellular degeneration, or cholestasis. Renal parenchyma remained structurally preserved beyond the localized perinephric findings, with intact glomerular tufts, proximal and distal tubular epithelium, and no signs of interstitial nephritis or tubular necrosis. Collectively, these observations indicate that repeated administration of GOX-loaded EVs is well tolerated, with only low-grade, primarily lymphoid-driven tissue responses at higher doses. Detailed morphological descriptions, scoring assessments, and representative micrographs for all organs and dose groups are provided in **Figure 3** and **Supplementary Table 2**. The pattern of alterations, including localized, non-destructive and immunologically coherent, suggests immune activation rather than classical toxicity. This distinction holds translational relevance: controlled modulation of lymphoid compartments may have implications for EV-mediated delivery systems in oncology, where engaging, reshaping, or leveraging immune microenvironments is often therapeutically desirable. The absence of structural injury in critical organs, including the liver, heart, kidneys, and lungs, further supports a favorable safety margin for potential clinical development of these GOX-loaded EV platforms.

### Immunotoxicity studies

**Figure 4:**
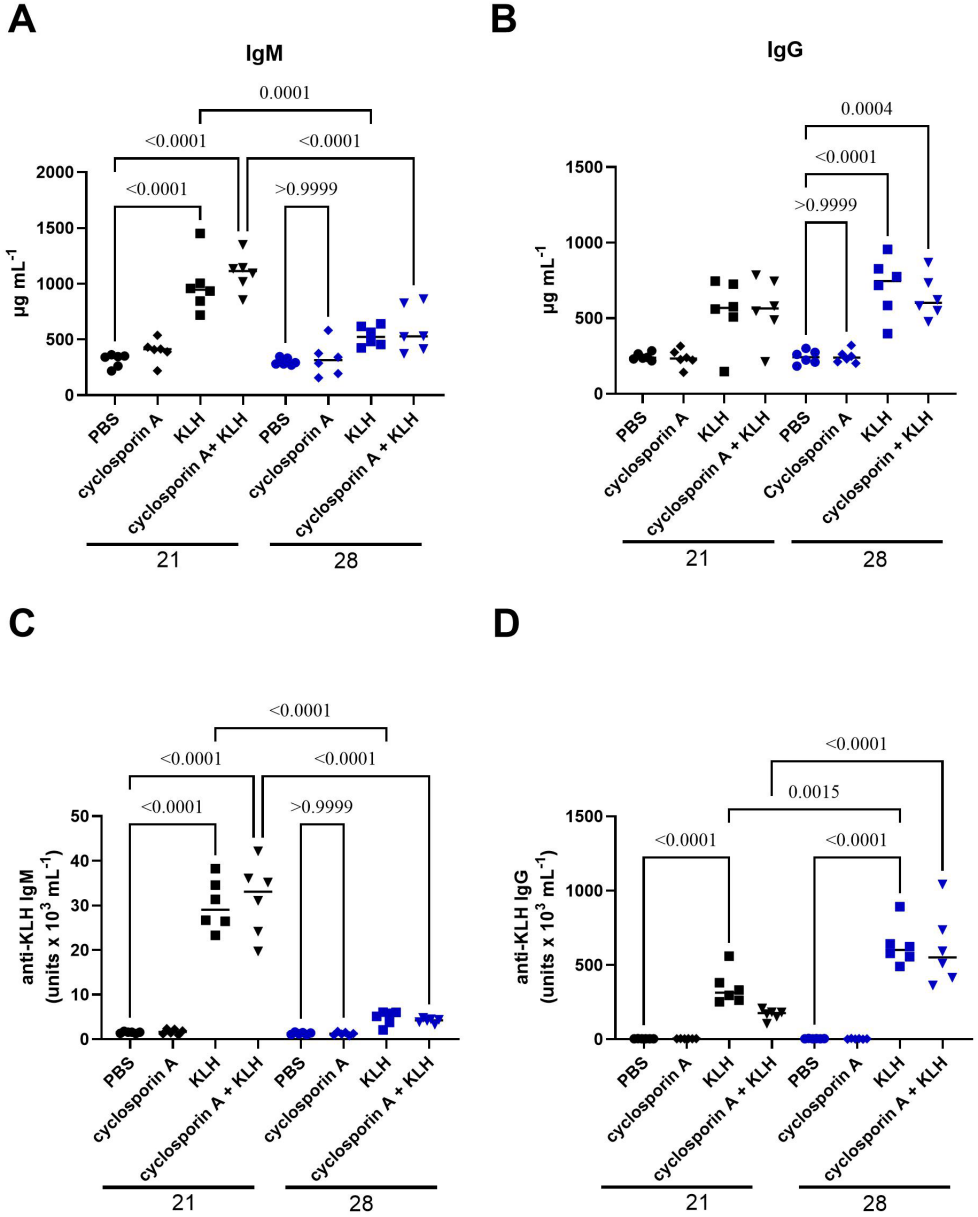
Validation of the immunotoxicity model using cyclosporin A in KLH-immunized BALB/c mice. Total serum IgM **(A)**, total serum IgG **(B)**, KLH-specific IgM **(C)**, and KLH-specific IgG **(D)** levels were measured on study days 21 and 28 in mice treated with vehicle control (PBS), cyclosporin A (positive immunosuppressive control), keyhole limpet hemocyanin (KLH), or a combination of cyclosporin A and KLH. KLH immunization was used to induce a defined antigen-specific humoral immune response, while cyclosporin A served as a reference control for systemic immunosuppression. KLH-specific antibody levels are expressed as arbitrary units, as described in the Materials and Methods. Data are presented as individual animals with mean ± SD. Statistical analysis was performed using ANOVA followed by Tukey’s multiple comparison test; significance bars indicate the specific group comparisons shown. Statistical significance is indicated by exact p-values provided in the images.

The TDAR assay provided a high-resolution functional readout of early and late humoral immunity, enabling quantitative discrimination between total immunoglobulins (IgM, IgG) and KLH-specific responses under a controlled T-cell-dependent antigen challenge. Throughout the study, GOX-loaded EVs were continuously administered, whereas KLH was delivered as a single immunization on day 14. Peripheral blood sampling on days 21 and 28 captured the transition from the extrafollicular response to the germinal-center (GC) maturation phase. By day 21, both the KLH and CsA-treated groups exhibited a synchronized induction of the primary humoral response, reflected by elevated total IgM and KLH-specific IgM titers. These kinetics are consistent with rapid plasmablast expansion driven by early BCR engagement and T-cell priming, preceding the establishment of mature GC structures. The subsequent decline in IgM levels by day 28 (**Figures 4A, B**) mirrors the physiologic contraction of the short-lived plasmablast pool and the transition toward class-switched antibody production. Total IgG concentrations peaked on day 28 following KLH immunization (**Figure 4B**), reflecting the culmination of GC-dependent class switching and affinity maturation. All animals generated detectable KLH-specific IgM and IgG responses; however, the KLH-specific IgG response remained comparatively modest (**Figures 4C, D**), suggesting either a restrained GC output or a limited duration of TFH-mediated help in this experimental framework. In the CsA treatment group, which was expected to attenuate T-cell–dependent B-cell activation, did not markedly suppress total IgM or IgG production after KLH exposure, nor did it significantly reduce KLH-specific IgM titers (**Figures 4A–C**). This pattern underscores the relative resistance of early extrafollicular responses to calcineurin inhibition. In contrast, KLH-specific IgG titers in CsA-treated animals were clearly diminished on day 21 (**Figure 4D**), aligning with CsA-mediated impairment of TFH function, GC seeding, and immunoglobulin class switching. Overall, KLH immunization elicited a >400-fold amplification of KLH-specific IgG relative to baseline and an approximately 30-fold increase in KLH-specific IgM by day 21 (**Figure 4D**).

**Figure 5:**
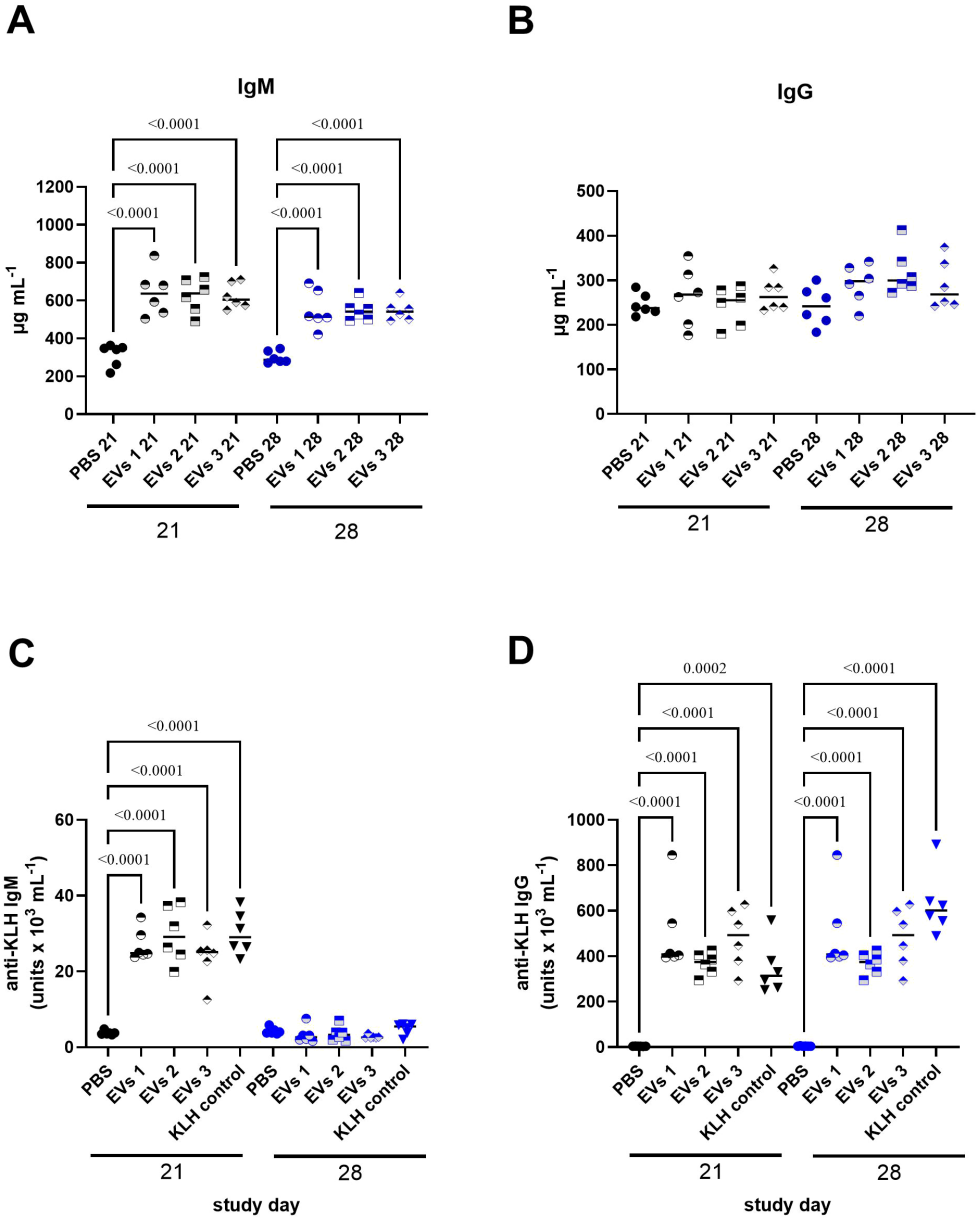
Effects of GOX-loaded extracellular vesicles on humoral immune responses following KLH immunization. Total serum IgM **(A)**, total serum IgG **(B)**, KLH-specific IgM **(C)**, and KLH-specific IgG **(D)** levels were measured on study days 21 and 28 in BALB/c mice treated with vehicle control (PBS), EVs (EVs 1–3), or KLH control, as described in the Materials and Methods. This experimental setup was designed to assess the immunomodulatory potential of EV treatment in the absence of pharmacological immunosuppression. KLH-specific antibody levels are expressed as arbitrary units. Data are shown as individual animals with mean ± SD indicated. Statistical analysis was performed using ANOVA followed by Tukey’s multiple comparison test; significance bars denote the specific comparisons indicated. Statistical significance is indicated by exact p-values provided in the images.

These findings delineate a robust yet temporally stratified TDAR response, characterized by intact extrafollicular IgM induction and a measurable, though partially constrained, GC-dependent IgG output. Such a profile offers a sensitive platform for detecting nuanced immunomodulatory effects of GOX-loaded EVs and calcineurin pathway inhibition on T-cell–dependent humoral immunity. The study evidenced that A549-derived GOX-loaded EVs elicited a pronounced activation of the early humoral arm, enhancing total IgM production across all three tested concentrations on both days 21 and 28. This selective amplification of IgM, in the absence of substantial IgG modulation (**Figures 5A, B**), is consistent with EV-driven potentiation of extrafollicular B-cell responses and short-lived plasmablast expansion. Although total IgG remained largely stable, a modest increase emerged on day 28, coinciding with the expected onset of germinal-center–dependent class switching. Under KLH stimulation, GOX-loaded EVs did not alter KLH-specific IgG titers, indicating limited impact on T-cell–dependent class-switch pathways, yet they significantly reduced KLH-specific IgM levels on day 28 (**Figures 5C, D**). This pattern suggests a shift in early B-cell activation thresholds or competitive modulation of antigen processing and presentation, resulting in attenuated primary IgM output without compromising downstream IgG maturation. Together, these data position tumor-derived EVs as active immunomodulators that preferentially rewire early antibody responses while sparing the germinal-center–derived IgG axis.

**Figure 6:**
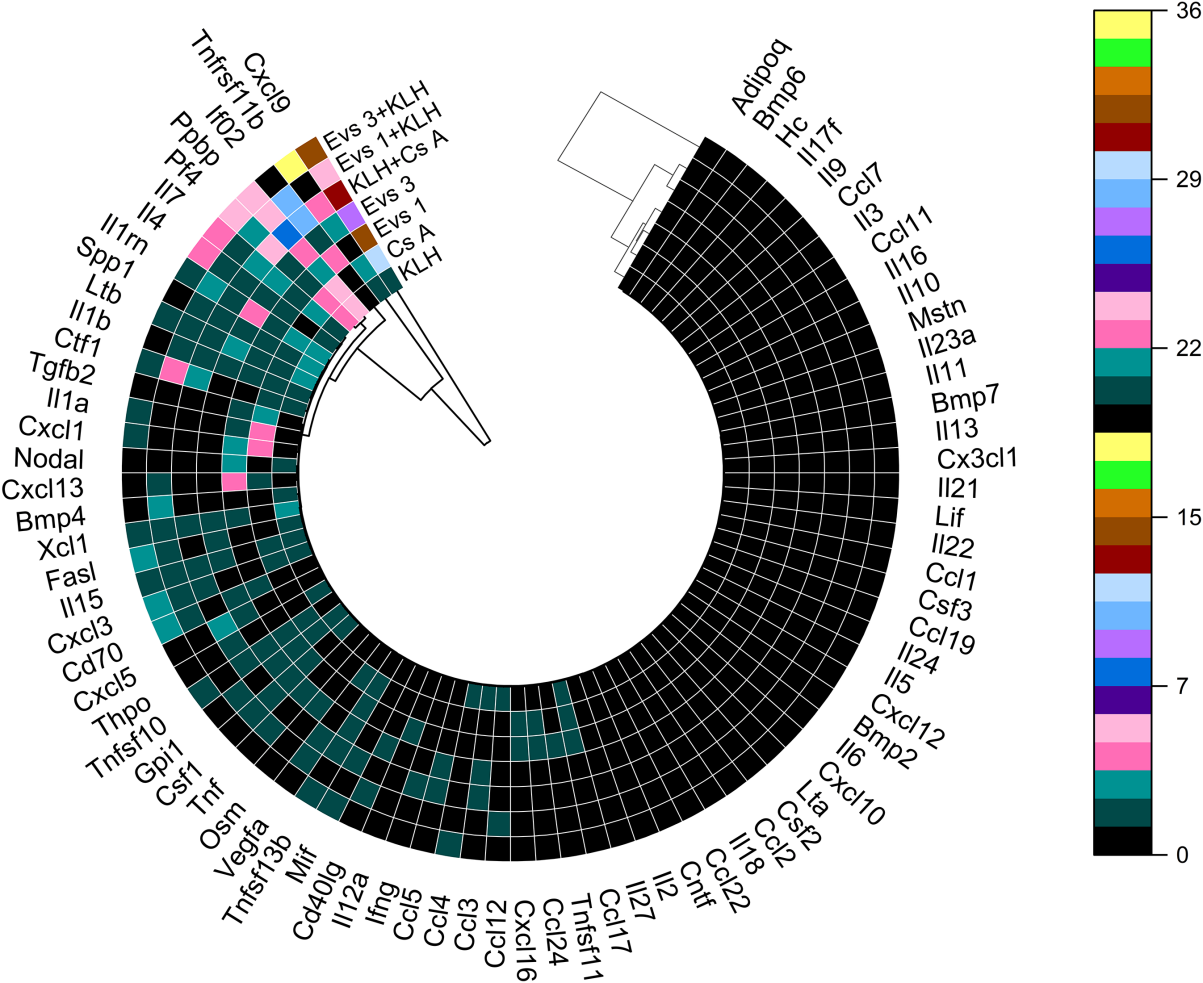
Expression of cytokine- and lymphokine-related genes in BALB/c mice following treatment with GOX-loaded extracellular vesicles and KLH immunization. Circular heatmap illustrates relative mRNA expression levels of selected cytokine and lymphokine genes measured in blood cells collected from BALB/c mice subjected to the indicated treatment conditions: phosphate-buffered saline (PBS, control), hemocyanin (KLH), cyclosporin A (CsA), GOX-loaded EVs at 10^4^ mL^-1^ (EVs 1), GOX-loaded EVs at 10^8^ mL^-1^ (EVs 3), KLH combined with CsA, KLH combined with EVs 1, and KLH combined with EVs 3. Gene expression data are presented as changes in mRNA levels relative to the control group (PBS-treated mice, group 1). Each radial segment corresponds to an individual gene, while concentric tracks represent different experimental groups. Hierarchical clustering with dendrogram visualization was applied to identify similarities in gene expression profiles across treatment conditions. Data were derived from n = 6 animals per group.

**Figure 7:**
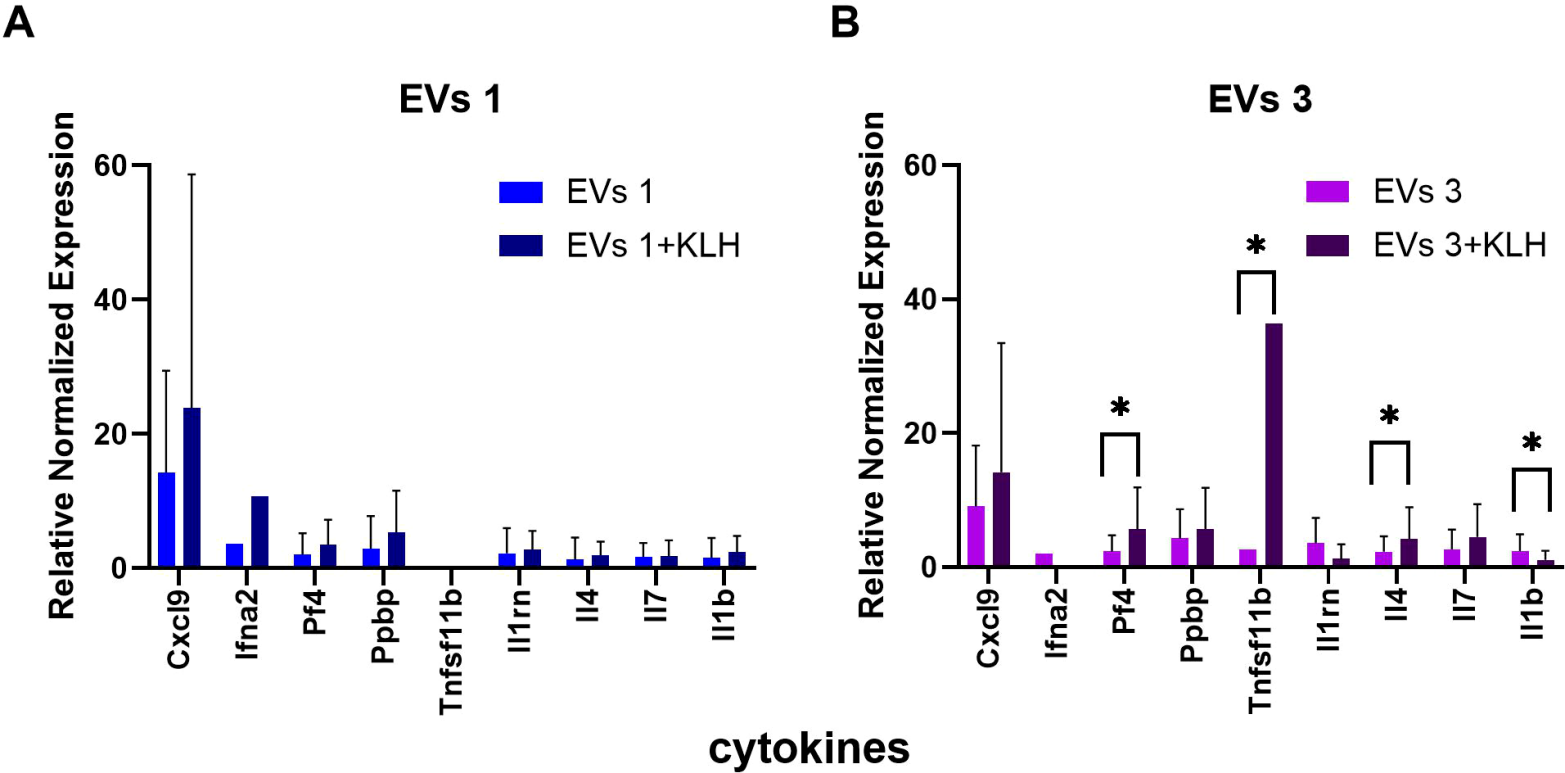
Relative expression of selected cytokine genes in peripheral blood cells following treatment with GOX-loaded extracellular vesicles and KLH immunization. **(A)** Relative mRNA expression levels of selected cytokines measured in peripheral blood cells from BALB/c mice treated with GOX-loaded EVs at 10^4^ mL^-1^ (EVs 1) or EVs 1 combined with keyhole limpet hemocyanin (EVs 1 + KLH). **(B)** Relative mRNA expression levels of the same cytokines measured in mice treated with GOX-loaded EVs at 10^8^ mL^-1^ (EVs 3) or EVs 3 combined with KLH (EVs 3 + KLH). Gene expression levels were normalized to the control group (PBS-treated mice) and are presented as relative normalized expression values. Data are shown as mean ± SD. Asterisks indicate statistically significant differences between EV-treated groups and the corresponding EV + KLH groups ( p < 0.05).*

Cytokine mRNA profiles were quantified in peripheral blood at the conclusion of the study to characterize the immunomodulatory effects of GOX-loaded EVs. A total of 82 gene targets were analyzed (**Figure 6**). Among all evaluated transcripts, A549-derived EVs that were loaded with GOX at both tested doses prominently modulated *Cxcl9*, a chemokine crucial for CXCR3-dependent T-cell trafficking, leukocyte differentiation, and tissue infiltration. This selective upregulation positions *Cxcl9* as the dominant chemokine axis engaged by EV exposure. A mild, dose-independent increase in *Cxcl3* and *Cxcl16* transcription was also detected. These chemokines, together with *Pf4* and *Ppbp*, are potent chemoattractants that cooperate in orchestrating inflammatory cell recruitment and amplifying innate immune activation. Subtle elevations were likewise observed for *Ccl5*, *Ccl12*, and *Ccl17*. Functionally, CCL5 (predominantly produced by T cells and monocytes) sustains chronic inflammatory tone; CCL12 promotes chemotaxis of eosinophils, monocytes, and lymphocytes; and CCL17 preferentially recruits Th2 cells, contributing to a range of immunoregulatory and inflammatory processes. Notably, the lower EV dose induced a significant upregulation of *Ifna2*, irrespective of KLH immunization status. IFNA2, a type I interferon, plays a pivotal antiviral, antiproliferative, and immunomodulatory role (**Figure 7A**). Its selective induction suggests that low-dose EV exposure may preferentially activate nucleic-acid-sensing pathways or modulate plasmacytoid dendritic cell-like transcriptional signatures. At the higher EV dose, modulation of *Tnfrsf11b*, a TNF receptor superfamily member encoded on chromosome 8, was evident, mirroring expression patterns observed in CsA-treated animals. This suggests that EVs may influence TNF-dependent signaling thresholds in a manner partially convergent with calcineurin pathway inhibition. GOX-loaded EVs also altered the expression of key pro-inflammatory interleukins, including *Il1rn*, *Il4*, and *Il7*, in a dose-dependent manner (**Figure 7B**). Together, these cytokines regulate inflammatory resolution (IL1RN), Th2 polarization (IL4), and lymphocyte survival and homeostasis (IL7), indicating that EVs exert broader immunoregulatory pressure on both innate and adaptive cytokine networks. Overall, these findings reveal a coherent cytokine response architecture dominated by chemokine signaling, subtle inflammatory tuning, and dose-dependent engagement of type I interferon pathways, highlighting a nuanced immunomodulatory capacity of tumor-derived GOX-loaded EVs.

## Discussion

In recent years, extracellular vesicles have emerged as clinically relevant therapeutic vectors, particularly in oncology, due to their intrinsic ability to transport pharmacologically active compounds, nucleic acids, and regulatory macromolecules with high biological specificity (46–48). Recent advances further highlighted EVs as multifunctional regulators of the tumor microenvironment, capable of simultaneously mediating drug delivery, immune modulation, and intercellular signaling reprogramming, positioning them at the forefront of next-generation biologics (49, 50). Their potential to modulate tumor-immune interactions and deliver molecular payloads directly into malignant cells positions EVs as a compelling next-generation platform for precision cancer therapy. Nevertheless, before their implementation in clinical practice, a rigorous toxicological and immunotoxicological characterization is imperative to ascertain their safety profile and prevent unintended host responses. EVs exhibit extensive biochemical and functional heterogeneity. Their molecular cargo, composed of context-dependent patterns of proteins, lipids, small RNAs, long non-coding RNAs, and DNA fragments, often posttranslationally modified, reflects both the lineage and activation state of the parental cells. Emerging single-vesicle and multi-omics studies demonstrate that even EV populations derived from a single tumor cell line can encompass functionally divergent subpopulations with distinct immunological and pharmacological effects (51, 52). In cancer, this heterogeneity becomes even more pronounced, as malignant cells release EVs enriched in oncogenic drivers, immune-modulatory ligands, and metabolic effectors. Such variability complicates the prediction of EV biodistribution, immunogenicity, and therapeutic behavior (53–55). From a translational perspective, understanding these molecular determinants is crucial to minimize off-target interactions and optimize therapeutic window. Despite their promise, EV-based therapeutics may provoke clinically relevant adverse effects. Recent reports have highlighted that EV-mediated immune modulation is highly context-dependent and dose-sensitive, ranging from immune tolerance induction to overt pro-inflammatory activation, depending on EV origin, cargo composition, and exposure schedule (56, 57). For instance, depending on their membrane proteome and cargo composition, EVs may engage pattern-recognition receptors, Fc-related pathways, or antigen-presenting cell networks, thereby triggering innate and adaptive immune activation that could manifest as systemic inflammation or autoimmune-like reactions (58). Likewise, EV-mediated transfer of microRNAs, transcriptional regulators, or metabolic enzymes may perturb essential signaling pathways in non-target tissues, interfering with normal cell homeostasis and potentially precipitating organ-specific toxicities (4, 55, 59, 60). Their pharmacokinetics further introduces an additional layer of complexity: after systemic or local administration, EVs may demonstrate preferential tropism toward specific organs, such as the liver, spleen, lungs, or kidneys, leading to bioaccumulation and localized toxicity, which is of high clinical relevance. Given these translational and safety considerations, regulatory agencies mandate comprehensive preclinical evaluation prior to the development of EV-based medicinal products (61, 62). In accordance with these requirements, the present study delineates acute and repeated-dose toxicity studies and immunotoxicity assessments of GOX-loaded EVs derived from A549 lung cancer cells performed under the OECD 407 guideline (63). The study design integrates advanced immunopharmacological principles based on the T-cell-Dependent Antibody Response (TDAR) paradigm, allowing for sensitive detection of EV-mediated modulation of humoral immunity. In parallel, the experimental framework adheres to the ICH S8 guideline on immunotoxicity evaluation of human pharmaceuticals (43), which provides regulatory standards for assessing unintended immunomodulatory effects, particularly for innovative biologics intended for oncological indications. To complement the systemic toxicological endpoints, comprehensive histopathological analyses were conducted on key organs, such as heart, lung, liver, kidney and spleen, collected at study termination. This organ-focused evaluation provides granular insights into potential EV-induced alterations in tissue architecture, local immune cell infiltration, microvascular integrity, and parenchymal function, collectively enabling a robust clinical-grade safety assessment of the engineered EV platform.

In the acute toxicity studies, no mortality, agonal states, or clinical signs necessitating early study termination were observed. Administration of the maximum tested dose of A549-derived GOX-loaded EVs did not produce any discernible toxic effects, aside from a statistically non-significant reduction in body weight recorded over the 15-day post-exposure period following the single administration. Furthermore, histopathological evaluation revealed no degenerative alterations in any of the examined organs from either control or EV-treated animals, and no inflammatory infiltrates were detected across the collected tissue samples. From a translational perspective, the absence of systemic, behavioral, or clinical toxicity across both short- and mid-term exposure windows provides a compelling foundation for advancing these EV formulations toward clinical-grade development. The maintained physiological and behavioral homeostasis suggests a low likelihood of off-target organ toxicity, a key determinant of safety in early-phase oncology therapeutics. Moreover, the preserved metabolic and neurological function *in vivo* aligns with the emerging perception of EV-based therapeutics as inherently biocompatible platforms capable of delivering bioactive cargo with reduced systemic burden compared to synthetic nanocarriers. These findings also support the feasibility of dose escalation strategies in future first-in-human studies and pave the way for more sophisticated pharmacokinetic and biodistribution analyses steps essential for integrating EV-based systems into precision oncology frameworks. In particular, the tolerability observed here may facilitate the development of EV-mediated delivery systems for targeted modulation of oncogenic signaling networks in lung cancer, while maintaining an acceptable therapeutic index required for clinical translation. From a regulatory perspective, the OECD Test Guideline 423 is designed to assess the hazard potential of the test article in its final form and does not require separate *in vivo* evaluation of individual components when the investigational product is a composite system. Accordingly, the comparison of the final EV-enzyme formulation to the vehicle alone is compliant with the guideline and addresses the primary regulatory question of acute toxicity of the administered product. From a scientific perspective, however, the absence of a parallel *in vivo* group receiving unloaded (empty) EVs limits the ability to fully deconvolute relative contributions of the EV carrier versus the enzymatic cargo to any observed effects. The present study design was informed by prior nonclinical *in vitro* data, which demonstrated that unloaded EVs originating from A549 cells formulated in PBS did not exhibit cytotoxicity across the tested cellular *in vitro* models, while the EV-enzyme formulation showed measurable toxicity *in vitro* under identical experimental conditions (21, 41). Based on these data, the EV carrier alone was considered to present a low intrinsic acute toxicity risk, whereas the enzymatic payload and/or its interaction with the EV system was identified as the primary driver of biological activity. Consequently, inclusion of an additional *in vivo* acute toxicity group consisting of unloaded EVs was assumed unlikely to provide incremental safety-relevant information at this stage. Consistent with the 3R principles (Replacement, Reduction, Refinement) embedded in OECD 423, the study design intentionally minimized animal use by limiting *in vivo* testing to groups necessary to address the primary regulatory safety question. The decision to exclude unloaded EVs from this acute study reflects a risk-based approach integrating existing *in vitro* evidence and ethical considerations, rather than an omission due to oversight. The resulting acute toxicity data should therefore be interpreted as characterization of the hazard profile of the final drug product, not as a standalone assessment of EV carrier safety. The study is considered supportive for early nonclinical development and dose selection rationale. We recognize that further differentiation between carrier-related and cargo-related effects may be required at later stages of development.

In the present study, in full alignment with current regulatory frameworks governing advanced biological therapeutics, a detailed and mechanistically oriented assessment of the immunotoxic potential of the investigated extracellular vesicles was undertaken. Immunogenicity profiling of EV-based products represents a critical component of preclinical safety evaluation, as EVs may interact with multiple layers of the immune system, including innate pattern-recognition pathways, antigen-presenting cell activation and downstream adaptive immune responses. The TDAR assay interrogates the integrity of T-cell-dependent humoral immunity by quantifying antigen-specific antibody synthesis, a process that integrates multiple immunological compartments, including dendritic cell-mediated antigen presentation, T-helper cell activation, germinal-center formation, and B-cell class-switch recombination. Because this cascade requires coordinated cross-talk between APCs, CD4^+^ T cells and B cells, deviations in antibody output against a defined antigen can reveal EV-induced perturbations across any of these immunological nodes. In this study, we employed KLH - a structurally complex, highly immunogenic neo-antigen - to generate a robust TDAR response, as KLH reliably induces high-titer antigen-specific antibodies through potent activation of follicular helper T cells and germinal-center B cells. In our experiments, KLH-specific IgM titers in EV-treated animals remained comparable to those in the control group on study days 21 and 28, indicating preserved early extrafollicular B-cell activation. Conversely, KLH-specific IgG titers were reduced relative to controls on study day 21. This divergence is consistent with established observations that the magnitude and immunophenotype of cyclosporine-dependent suppression in TDAR systems are highly sensitive to experimental variables, such as KLH dose, route of immunization and timing of serum collection. Nevertheless, a stable immunological principle persists: IgG responses reflecting germinal-center activity, Tfh-cell support, and class-switch recombination are considerably more susceptible to suppression than IgM (64). This differential sensitivity indicates that IgG production serves as a more responsive biomarker for disruptions in T-cell-mediated cytokine networks and CD40L-CD40-dependent B-cell maturation pathways (64, 65). Consistent with these mechanisms, prior studies have likewise documented minimal cyclosporine-mediated inhibition of KLH-specific IgM (65). After approximately four weeks of repeated administration of GOX-loaded EVs at three dose levels, KLH-immunized animals exhibited a significant reduction in KLH-specific IgM titers. Notably, in non-immunized animals, EV treatment increased total IgM concentrations, suggesting that the GOX-loaded EVs themselves possess intrinsic immunogenic properties capable of activating naïve or marginal-zone B-cell populations. Following KLH immunization, EV exposure enhanced total IgM and IgG levels but did not alter KLH-specific IgM or IgG synthesis. The transient elevation of total IgM, together with reduced antigen-specific IgM, is consistent with low-grade, non-clonal B-cell activation, a phenomenon well described in the context of innate immune cues, metabolic stress, or altered redox environments. Importantly, such responses are typically dominated by short-lived, polyreactive IgM secretion and are not inherently adverse, provided that antigen-specific responses and class switching are preserved - as is the case in our study. This also supports the interpretation that the EVs induce broad immunostimulation rather than antigen-focused humoral amplification. Continuous exposure to EV-associated membrane proteins, lipids and potential DAMP-like molecular signatures, unlike the single-bolus antigenic challenge provided by KLH may have preferentially driven immunoglobulin synthesis toward epitopes presented by the EVs themselves. Thus, A549-derived GOX-loaded EVs appear to function as subtle molecular immunomodulators, enhancing basal immune activation through low-grade stimulation of APCs and B-cell subsets, rather than amplifying the canonical germinal-center response to KLH. This immunological dissociation between polyclonal immunoglobulin induction and antigen-specific responses is increasingly recognized as a hallmark of EV-mediated immune conditioning rather than classical adjuvanticity (50, 66–68). The TDAR findings, therefore, demonstrate that repeated administration of A549-derived EVs induces a modest but biologically detectable systemic immune activation. This is further aligned with the histopathological evaluation, which revealed only minimal lesions across all examined organs. It should be emphasized that tumor-derived extracellular vesicles (TEVs) are traditionally described as contributors to immune evasion, metastatic niche formation, and suppression of antitumor immunity. However, emerging evidence highlights that engineered or compositionally altered TEVs can acquire immunostimulatory functions under specific conditions. Our findings - characterized by selective IgM amplification, preserved antigen-specific IgG responses, and absence of systemic inflammation - align with this evolving view and warrant deeper consideration. A growing body of work, exemplified by Yang *et al.* (69), shows that tumor-cell-derived membrane materials, including TEVs, can be repurposed as potent immunogenic platforms within cancer vaccines. The authors emphasizes that tumor cell membranes and TEVs inherently carry a broad repertoire of tumor-associated antigens and can act as integrated carriers for adjuvants or therapeutic molecules, thereby promoting immune recognition rather than tolerance when presented in appropriately engineered formats. Notably, they describe how modifications such as membrane hybridization, adjuvant incorporation, and physicochemical re-engineering can shift TEVs from immunosuppressive mediators to initiators of productive antitumor immune responses. This perspective is directly relevant to our findings: although our EVs were not intentionally surface-engineered to enhance immunogenicity, the procedure of enzymatic cargo loading - combined with intrinsic tumor-antigen content - may have subtly altered their immunological behavior. Parallel insights can be drawn from recent advances summarized by Jiramonai *et al.* (70), who described and discussed EV-based strategies for tumor immunotherapy. Their analysis highlights that EVs possess unique immunomodulatory properties capable of counteracting tumor-induced immune tolerance, particularly by influencing antigen presentation, T-cell activation, and remodeling of the tumor microenvironment. Critically, EVs carrying specific molecular cues can enhance immune activation by overcoming immune-checkpoint-mediated suppression and stimulating innate or adaptive pathways. Although our study did not incorporate immune-activating ligands or checkpoint inhibitors, the observed elevation in total IgM without perturbation of KLH-specific IgG suggests a mild, nonspecific activation of innate-like B-cell compartments or marginal-zone B-cell subsets, which are known to respond preferentially to metabolic stress signals, redox imbalance, or non-classical danger cues without disrupting T-cell-dependent antibody maturation. This interpretation is further supported by the intact KLH-specific IgG response, indicating preserved germinal-center function and functional T-cell help. Importantly, while the EVs used in the present study were not intentionally engineered to elicit immunostimulation, the introduction of enzymatically active GOX represents a biologically meaningful alteration of EV cargo. Localized glucose oxidation and hydrogen peroxide generation are known to modulate immune cell activation threshold and may contribute to subtle immune sensing without triggering systemic inflammation. In this context, GOX-loaded tumor-derived EVs may act as a weak metabolic adjuvant, amplifying basal humoral activity while avoiding the immunotoxic profiles typically associated with strong innate immune agonists. The observed effects do not constitute evidence of antigen-specific immune targeting or therapeutic immunomodulation. Rather, they suggest that minimally engineered tumor-derived EVs can elicit detectable but limited immune engagement without compromising adaptive immune competence. This distinction is essential and aligns with current recommendations in the EV immunotherapy field to differentiate between immune activation, immune redirection, and immune toxicity.

Cytokines constitute a central regulatory axis of innate and adaptive immunity and serve as sensitive indicators of immunological and immunotoxic perturbations. In our study, exposure to GOX-loaded EVs robustly up-regulated the chemokine Cxcl9 across both administered doses (71). The lower dose preferentially activated a transcriptional program dominated by chemotactic mediators Pf4, Ppbp, Cxcl3, Cxcl16, CCL5, CCL12, and CCL17 (72–74). Functionally, these chemokines converge on signaling pathways orchestrating early leukocyte recruitment through G-protein-coupled receptor (GPCR) activation, leading to downstream PI3K-Akt, Rho-GTPase, and MAPK signaling. This cascade promotes cytoskeletal rearrangement, firm adhesion, and directed migration of distinct immune cell subsets. For example, Cxcl11 directs activated T-cell trafficking (71), Cxcl3 governs monocyte adhesion and extravasation (75), Cxcl16 supports homing of T cells and NKT cells (76), PF4 enhances neutrophil and fibroblast chemoattraction (77), and Ppbp modulates stromal activation by inducing mitogenesis, extracellular matrix deposition, and plasminogen activator synthesis (Ppbp) (78). At higher EV concentrations, a more complex immunological phenotype emerged, with increased expression of interleukins 1b, 4, and 7 (79–82) and TNF receptor superfamily members Tnfrsf11b and Tnfrs10 (83). TNF receptor proteins can function as decoy receptors, neutralizing TNF-family ligands and attenuating TNFR1-mediated apoptotic and necroptotic pathways, typically propagated via TRADD, FADD, and caspase-8. In contrast, up-regulated interleukins potentiate activation of canonical inflammatory transcriptional programs. IL-1β, through MyD88-IRAK4-TRAF6-TAK1, strongly induces NF-κB and AP-1, initiating a broad inflammatory gene signature encompassing adhesion molecules, secondary cytokines, and chemokines. This creates a feed-forward loop reinforcing leukocyte recruitment and endothelial activation. IL-4 primarily signals via JAK1/JAK3-STAT6, driving transcriptional programs characteristic of type-2 immunity, including differentiation of naïve CD4^+^ T cells into Th2 subsets, class switching of B cells, and enhanced antibody production (84, 85). IL-7, essential for lymphoid homeostasis, signals through JAK1/JAK3-STAT5, providing survival cues to early B- and T-cell progenitors by inducing Bcl-2 family-mediated anti-apoptotic pathways (86). The induction of IL-7 suggests activation of early lymphopoietic circuits, consistent with enhanced lymphoid expansion observed histologically. Overall, the combined cytokine profile reflects a coordinated activation of NF-κB-driven innate inflammation together with JAK-STAT-mediated adaptive immune priming. Such immune architectures have recently been implicated in enhancing antitumor immune surveillance and improving the efficacy of combination immunotherapies based on EV platforms (50, 56, 68, 87, 88). The chemokine milieu promotes rapid recruitment of monocytes, neutrophils, T cells, and NKT cells, while interleukin induction simultaneously expands and stabilizes lymphocyte populations. Such dual-layer activation is characteristic of evolving immune responses transitioning from innate initiation toward adaptive amplification. These transcriptional changes align closely with histopathological findings. At early exposure levels, tissue sections revealed prominent lymphoid follicular hyperplasia with active germinal centers and chronic perivascular inflammatory infiltrates, indicative of sustained antigenic stimulation. The presence of germinal centers corresponds to STAT6- and NF-κB-dependent B-cell activation, while perivascular infiltrates reflect persistent chemokine-driven trafficking of lymphocytes and myeloid cells. Altogether, the molecular and morphological data demonstrate that bioengineered EVs elicit a multilayered immune activation architecture, integrating chemotactic signaling with cytokine-driven lymphocyte proliferation and differentiation.

We are aware that integrating protein-level and functional cytokine assays would further strengthen the immunological interpretation of our findings. Our study was designed as an initial *in vivo* safety and immunotoxicity screen, in which transcript-level analysis was selected for two reasons. First, to enable broad, multiplexed profiling across multiple cytokine families from limited tissue material, and second, to detect early upstream immune signaling events that often precede measurable changes at the protein level. mRNA-based readouts are widely used in exploratory immunotoxicity assessments for this purpose. Importantly, the transcriptional data in our study revealed no coherent pattern of pro-inflammatory activation, no induction of canonical cytokine storm signatures, and no changes consistent with disruption of T-cell or innate immune homeostasis. These results, combined with the absence of clinical or histopathological abnormalities, support the conclusion that GOX-loaded EVs do not trigger overt inflammatory responses within the tested dose range. Nevertheless, we fully acknowledge that mRNA measurements alone cannot definitively confirm cytokine activity, as post-transcriptional regulation, secretion dynamics, and bioactive concentration at the protein level are essential for comprehensive immune interpretation. We therefore agree that the mechanistic depth of our cytokine assessment is limited.

**Figure 8:**
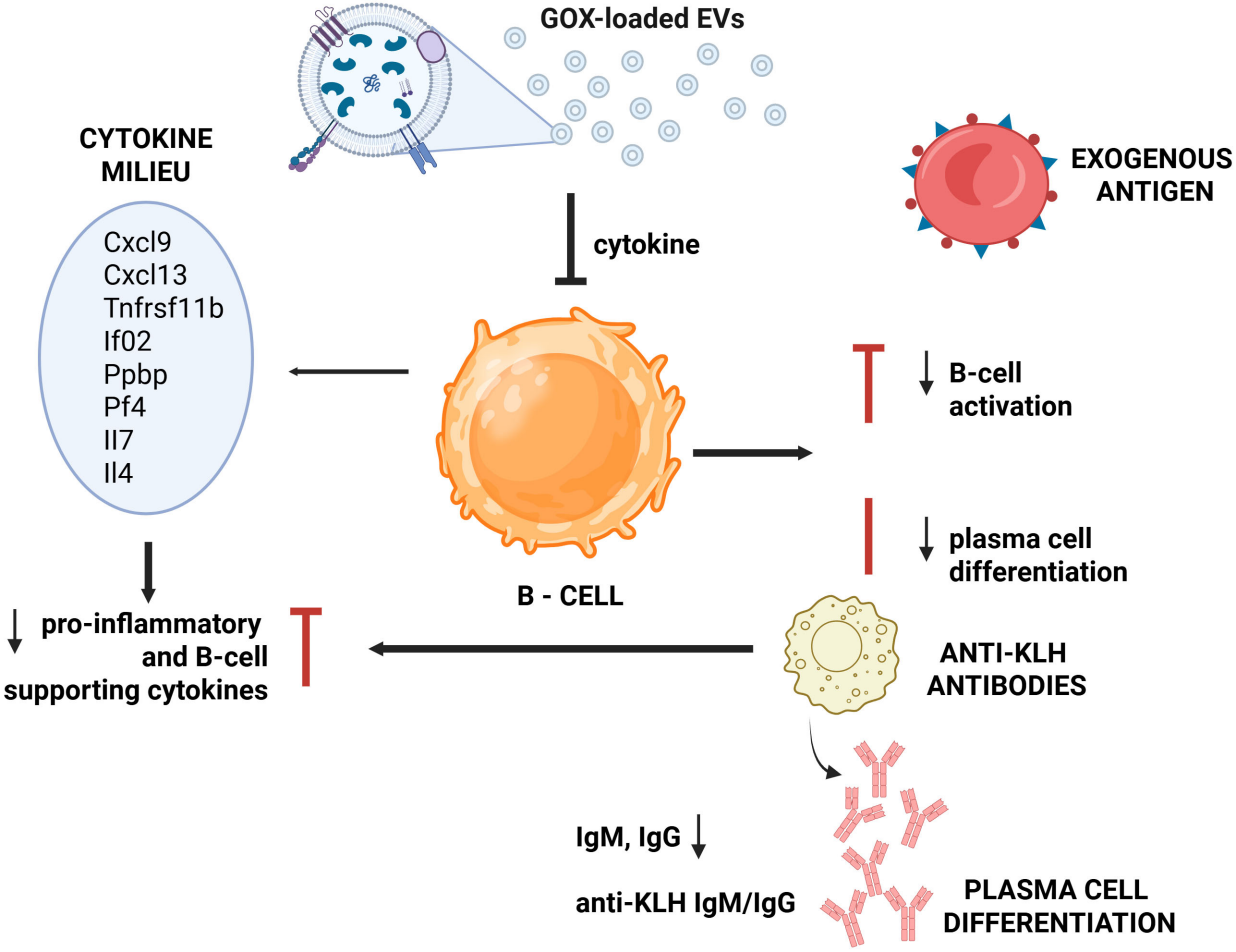
Schematic representation of the proposed mechanism of immunomodulation mediated by GOX-loaded EVs based on the TDAR study performed in BALB/c mice. The figure illustrates the impact of GOX-loaded extracellular vesicles (GOX-loaded EVs) on humoral immune responses and the associated cytokine-chemokine milieu required for effective B-cell activation and differentiation. Following *in vivo* administration, GOX-loaded EVs derived from A549 lung cancer cells interact with the immune system in a constrained and context-dependent manner, without inducing a classical inflammatory response. GOX-loaded EVs elicit a selective chemokine-biased response, characterized by upregulation of Cxcl9 and modest modulation of Cxcl3, Cxcl16, Ccl5, Ccl12, and Ccl17, in the absence of broad cytokine induction. Therefore, B-cell activation and differentiation into antibody-secreting plasma cells remain limited, leading to reduced production of antigen-specific antibodies. At the systemic level, humoral immune analysis reveals an increase in total circulating IgM at later time points, while IgG levels remain unchanged, indicating selective engagement of early humoral response elements without full isotype class switching. GOX-loaded EVs act as immunomodulatory entities, eliciting a selectively confined humoral response and a chemokine-biased immune profile, rather than exhibiting the broad immunostimulatory activity characteristic of classical immune adjuvants.

In conclusion, the present study comprehensively evaluated the acute and repeated-dose toxicity and the immunotoxicological profile of glucose oxidase-loaded extracellular vesicles derived from A549 lung cancer cells. The analytical framework applied herein enabled a systematic assessment of functional endpoints within the adaptive immune compartment, particularly T-cell-dependent antibody responses. The resulting dataset yields essential insights into the immunological safety profile of the investigated material, enabling evaluation of risks associated with excessive inflammatory signaling, complement activation, or unintended immune stimulation. These considerations are particularly pertinent for EV-based therapeutic platforms, given that their complex molecular cargo and their surface molecular signatures may differentially influence immune tolerance or immunoreactivity. Accordingly, the findings support a robust, mechanism-informed assessment of potential adverse immune events and facilitate the distinction between physiological murine host-GOX-loaded EV interactions and maladaptive or pro-inflammatory immune responses (**Figure 8**). Taken together, the presented immunogenicity evaluation establishes a scientifically rigorous foundation for subsequent stages of preclinical development. Furthermore, it informs the rational design of future investigational pathways, including early-phase clinical studies, by integrating immunological endpoints into a comprehensive risk-benefit evaluation framework. This strategy ensures that the systemic and immunobiological properties of the EVs are thoroughly characterized prior to human exposure, in alignment with current regulatory expectations for the development of innovative biological therapeutics.

## Data Availability

The raw data supporting the conclusions of this article will be made available by the authors, without undue reservation.
