# Supplementary material for: Systemic toxicity and immunotoxicity studies of glucose oxidase-loaded extracellular vesicles derived from lung cancer cells
Source: Front Immunol. 2026 May 11;17:1842887. doi: 10.3389/fimmu.2026.1842887 (PMC13199250; doi:10.3389/fimmu.2026.1842887)
Supplement: Supplementary file 1 [file SupplementaryFile1.docx]

**Supplementary Information**

**Systemic toxicity and immunotoxicity studies
of glucose oxidase-loaded extracellular vesicles
derived from lung cancer cells**

Ireneusz P. Grudzinski^1*^, Magdalena Bamburowicz-Klimkowska^1*^, Barbara Sochanowicz^2^, Kamil Brzoska^2^, Monika Prochorec-Sobieszek^3^, Marzena Cabaj^3^, Alicja Targonska^4^, Agnieszka Stawarska^1^ and Marcin Kruszewski^2,5*^

^1^ Medical University of Warsaw, Faculty of Pharmacy, Department of Toxicology and Food Science, 1 Banacha Str., PL-02-097 Warsaw, Poland

Pasteura Str 3, 02-093 Warsaw, Poland

^2^ Institute of Nuclear Chemistry Technology, Centre for Radiobiology and Biological Dosimetry, 16 Dorodna Str., PL-03-195 Warsaw, Poland

^3^ Institute of Hematology and Transfusion Medicine, 14 Indiry Gandhi Str., PL-02-776 Warsaw, Poland

^4^ Nencki Institute of Experimental Biology, Polish Academy of Sciences, 3 Pasteur Str., PL-02-093 Warsaw, Poland

^5^ Institute of Rural Health, Department of Molecular Biology and Translational Research, 2 Jaczewskiego Str., PL-20-090, Lublin, Poland

*Corresponding author: [ireneusz.grudzinski@wum.edu.pl](mailto:ireneusz.grudzinski@wum.edu.pl); [mjbamburowicz@wum.edu.pl](mailto:mjbamburowicz@wum.edu.pl); [m.kruszewski@ichtj.waw.pl](mailto:m.kruszewski@ichtj.waw.pl)


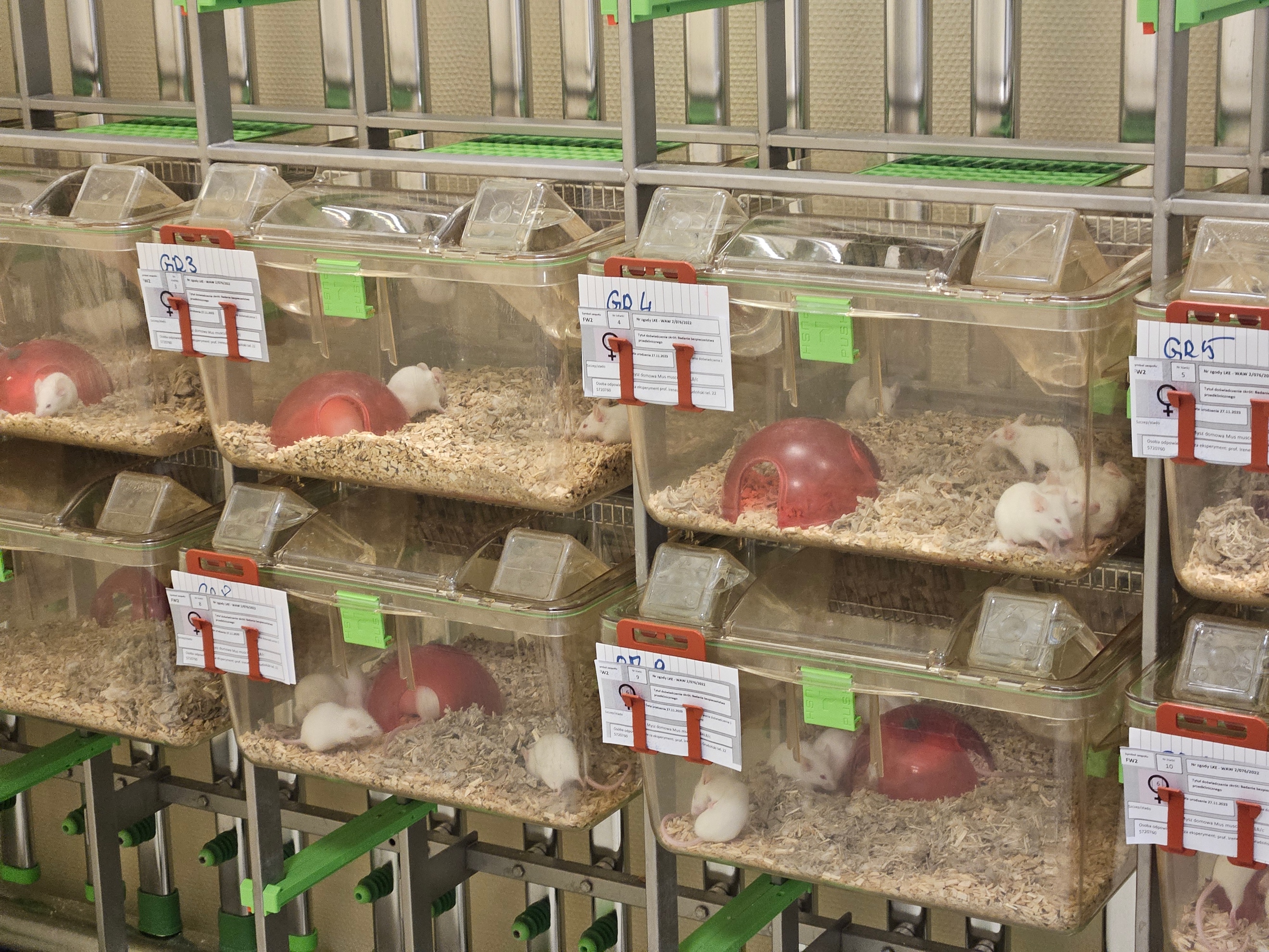


**Figure S1.** Individually ventilated cages (IVC), rack-type system with mechanical ventilation, equipped with air supply and exhaust units. BALB/c mice housed in the cages and used in preclinical safety (TDAR) studies. Animal facility of the Centre for Preclinical Studies, Medical University of Warsaw, Poland.

**Table 1**. **Representative resection specimens of female BALB/c mice subjected to glucose oxidase (GOX)-loaded extracellular vesicles (EVs) based on the acute toxicity study (H&E staining, original magnification ×200).** Inflammatory infiltrates (grade): 0 - none, 0-1 - minimal, 1 - small degree, 2 - medium degree. Hyperplasia of the white pulp of the spleen (grade); 0 - none, 0-1 - minimal, 1 - low degree, 2 - medium degree.

|  | Heart | Lung | Liver | Kidney | Spleen |
| --- | --- | --- | --- | --- | --- |
| Control group | 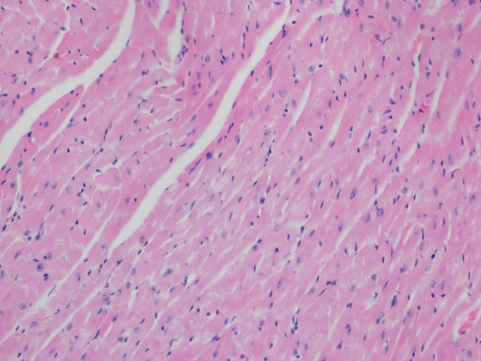  Microscopic image of normal myocardial tissue. No necrosis, degenerative changes, fibrosis, hypertrophy and inflammatory infiltrates. Inflammatory infiltrates (0). | 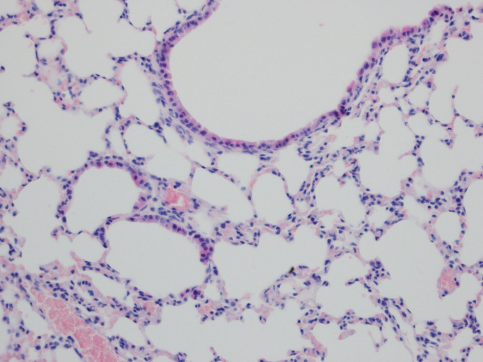  Microscopic image of the normal lung tissue. No necrosis, degenerative changes, alveolar damage, emphysema, fibrosis and inflammatory infiltrates. Inflammatory infiltrates (0). | 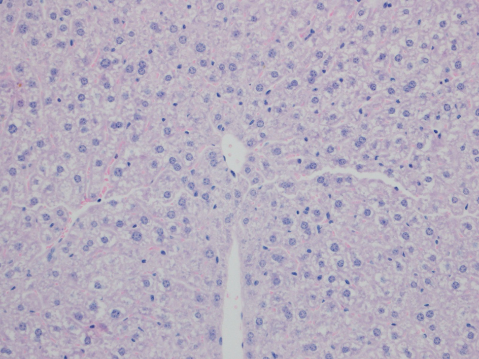  Microscopic image of the normal liver tissue. No necrosis, degenerative changes, bile stasis, fibrosis and inflammatory infiltrates. Inflammatory infiltrates (0). | 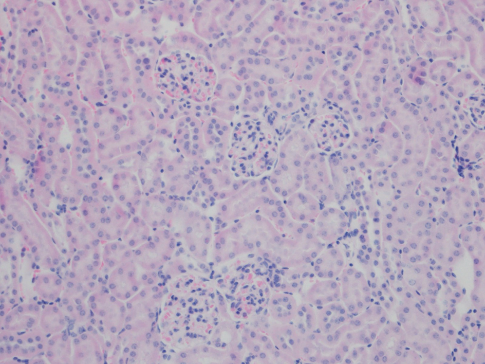  Microscopic image of the normal kidney tissue. No necrosis, degenerative changes, fibrosis and inflammatory infiltrates. Inflammatory infiltrates (0). | 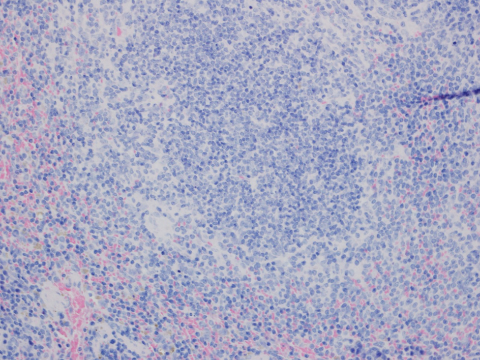  Microscopic image of normal spleen tissue. No necrosis, degenerative changes, congestion, fibrosis and white pulp hyperplasia (0). |
| EVs (10^8^ mL^-1^) treated group | 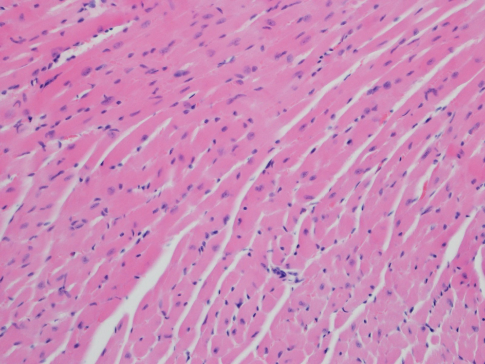  Microscopic image of normal myocardial tissue. No necrosis, degenerative changes, fibrosis, hypertrophy and inflammatory infiltrates. Inflammatory infiltrates (0). | 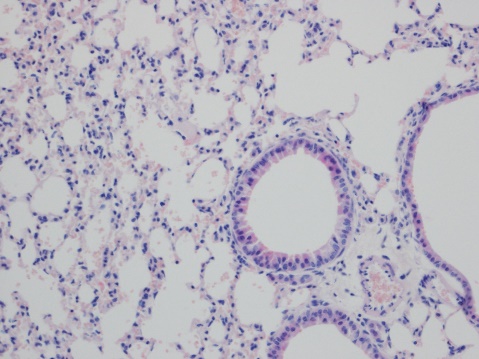  Microscopic image of the normal lung tissue. No necrosis, degenerative changes, alveolar damage, emphysema, fibrosis and inflammatory infiltrates. Inflammatory infiltrates (0) | 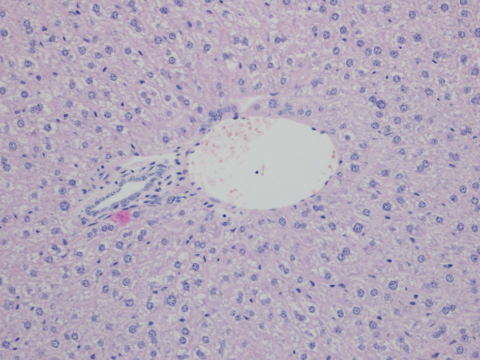  Microscopic image of the normal liver tissue. No necrosis, degenerative changes, bile stasis, fibrosis and inflammatory infiltrates. Inflammatory infiltrates (0). | 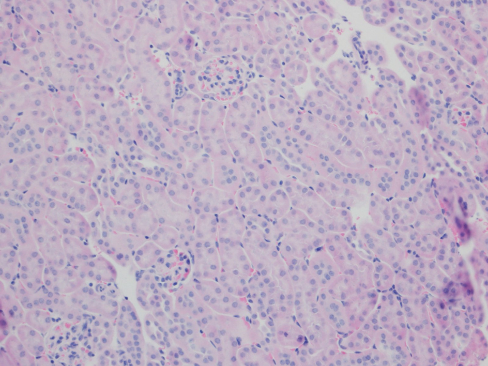  Microscopic image of the normal kidney tissue. No necrosis, degenerative changes, fibrosis or inflammatory infiltrates. Inflammatory infiltrates (0). | 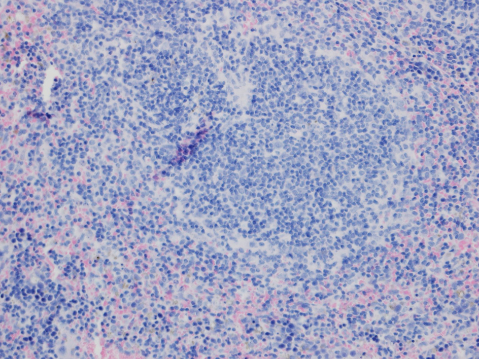  Microscopic image of normal spleen tissue. No necrosis, degenerative changes, congestion, fibrosis and white pulp hyperplasia (0). |

**Table 2**. **Representative resection specimens of female BALB/c mice subjected to glucose oxidase (GOX)-loaded extracellular vesicles (EVs) based on the repeated dose (28-days) toxicity study (H&E staining, original magnification ×200).** Inflammatory infiltrates (grade): 0 - none, 0-1 - minimal, 1 - small degree, 2 - medium degree. Hyperplasia of the white pulp of the spleen (grade); 0 - none, 0-1 - minimal, 1 - low degree, 2 - medium degree.

| Group | Heart | Lung | Liver | Kidney | Spleen |
| --- | --- | --- | --- | --- | --- |
| Group 1  Control (PBS) | 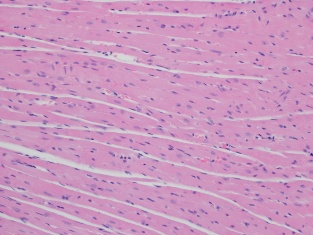  Microscopic image of normal myocardial tissue. No necrosis, degenerative changes, fibrosis, hypertrophy and inflammatory infiltrates. Inflammatory infiltrates (0). | 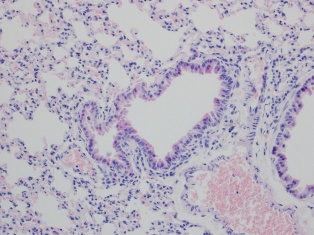  Microscopic image of the normal lung tissue. No necrosis, degenerative changes, alveolar damage, emphysema, fibrosis and inflammatory infiltrates. . Inflammatory infiltrates (0). | 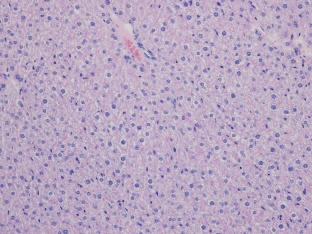  Microscopic image of the normal liver tissue. No necrosis, degenerative changes, bile stasis, fibrosis and inflammatory infiltrates. . Inflammatory infiltrates (0). | 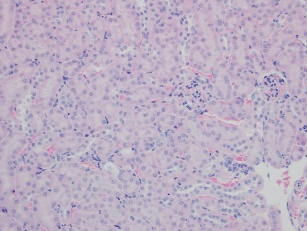  Microscopic image of the normal kidney tissue. No necrosis, degenerative changes, fibrosis or inflammatory infiltrates. Inflammatory infiltrates (0). | 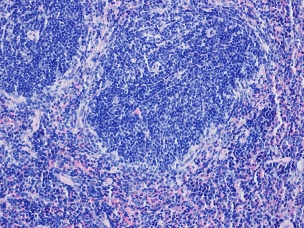  Microscopic image of normal spleen tissue. No necrosis, degenerative changes, congestion, fibrosis and white pulp hyperplasia (0). |
| Group 2  KLH hemocyanin  (immunostimulation) | 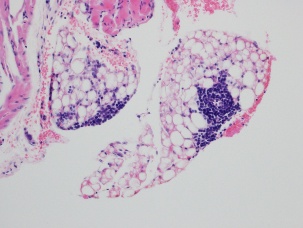  Myocardial fibers without microscopic changes. Small perivascular inflammatory infiltrates of chronic type (lymphocytes+ sparse plasma cells) in myocardium and pericardial fatty tissue. Inflammatory infiltrates (1). | 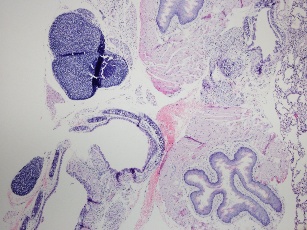  Alveoli and interstitial tissue without changes. Inflammatory infiltrates of chronic type with formation of secondary lymph nodules around bronchi and pulmonary bronchioles and sparse blood vessels.  Inflammatory infiltrates (2). | 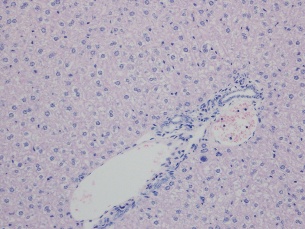  Hepatocytes without changes. Minimal inflammatory infiltrates of chronic type (lymphocytes) in portal-biliary spaces.  Inflammatory infiltrates (0-1). | 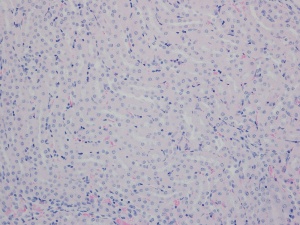  Glomeruli and renal tubules without changes.  Minimal inflammatory infiltrates of the chronic type (lymphocytes) in the interstitial tissue of the kidney.  Inflammatory infiltrates (0-1). | 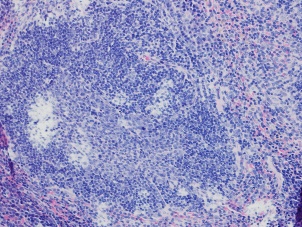  White pulp hyperplasia of the spleen. Lymhoid follicles with germinal centers (1). |
| Group 3  CSA cyclosporine A  (immunosuppression) | 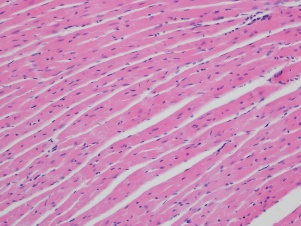  Microscopic image of normal myocardial tissue, without inflammatory infiltrates. Inflammatory infiltrates (0). | 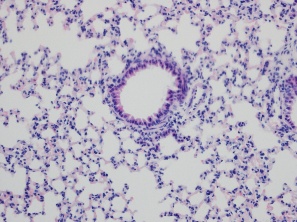  Microscopic image of normal lung tissue, without inflammatory infiltrates. Inflammatory infiltrates (0). | 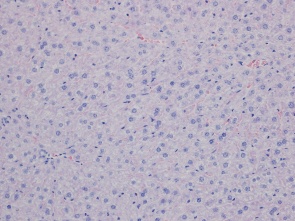  Microscopic image of normal liver tissue, without inflammatory infiltrates. Inflammatory infiltrates (0). | 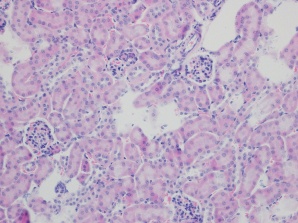  Microscopic image of normal kidney tissue, without inflammatory infiltrates. Inflammatory infiltrates (0). | 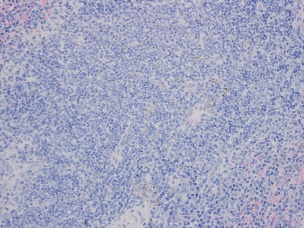  Slight reduction in the number and size of the lymphoid follicles of the white pulp of the spleen (0-1). |
| Group 4  KLH hemocyanin  (immunostimulation  +  CSA cyclosporine A  (immunosuppression | 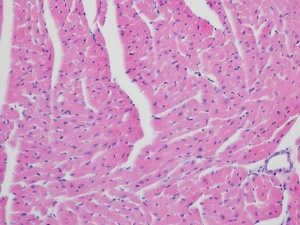  Microscopic image of normal myocardial tissue, without inflammatory infiltrates. Inflammatory infiltrates (0). | 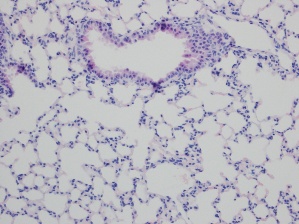  Microscopic image of normal lung tissue, without inflammatory infiltrates. Inflammatory infiltrates (0). | 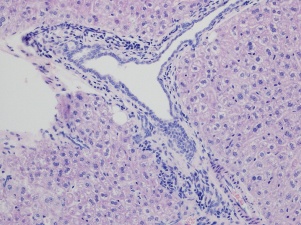  Hepatocytes without changes. Minimal inflammatory infiltrates of chronic type (lymphocytes) in portal-biliary spaces.  Inflammatory infiltrates (0-1). | 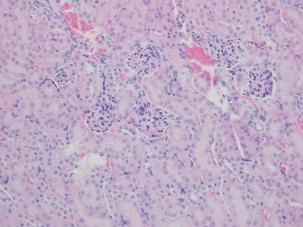  Microscopic image of normal kidney tissue, without inflammatory infiltrates. Inflammatory infiltrates (0). | 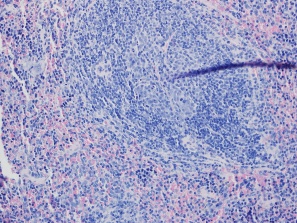  Slight hyperplasia of the white pulp of the spleen. Lymphoid follicles with germinal centers (0-1). |
| Group 5  EVs (Dose 1) | 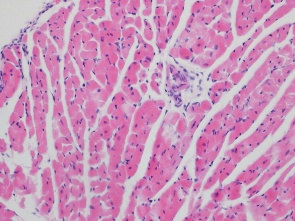  Myocardial fibers without microscopic changes. Minimal perivascular inflammatory infiltrates of the chronic type in the myocardium . Inflammatory infiltrates (0-1). | 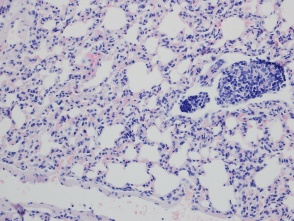  Alveoli and interstitial tissue without changes. Small inflammatory infiltrates of the chronic type around the pulmonary bronchioles. Inflammatory infiltrates (0-1). | 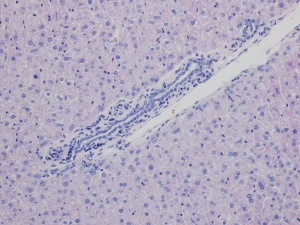  Hepatocytes without changes. Minimal inflammatory infiltrates of chronic type in portal-biliary spaces. Inflammatory infiltrates (0-1). | 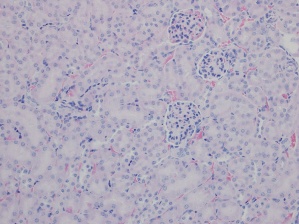  Microscopic image of normal kidney tissue, without inflammatory infiltrates. Inflammatory infiltrates (0). | 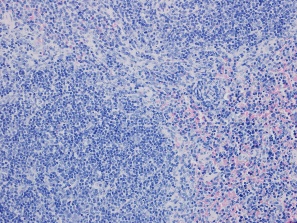  Microscopic image of a normal splenic tissue (0). |
| Group 6  EVs (Dose 2) | 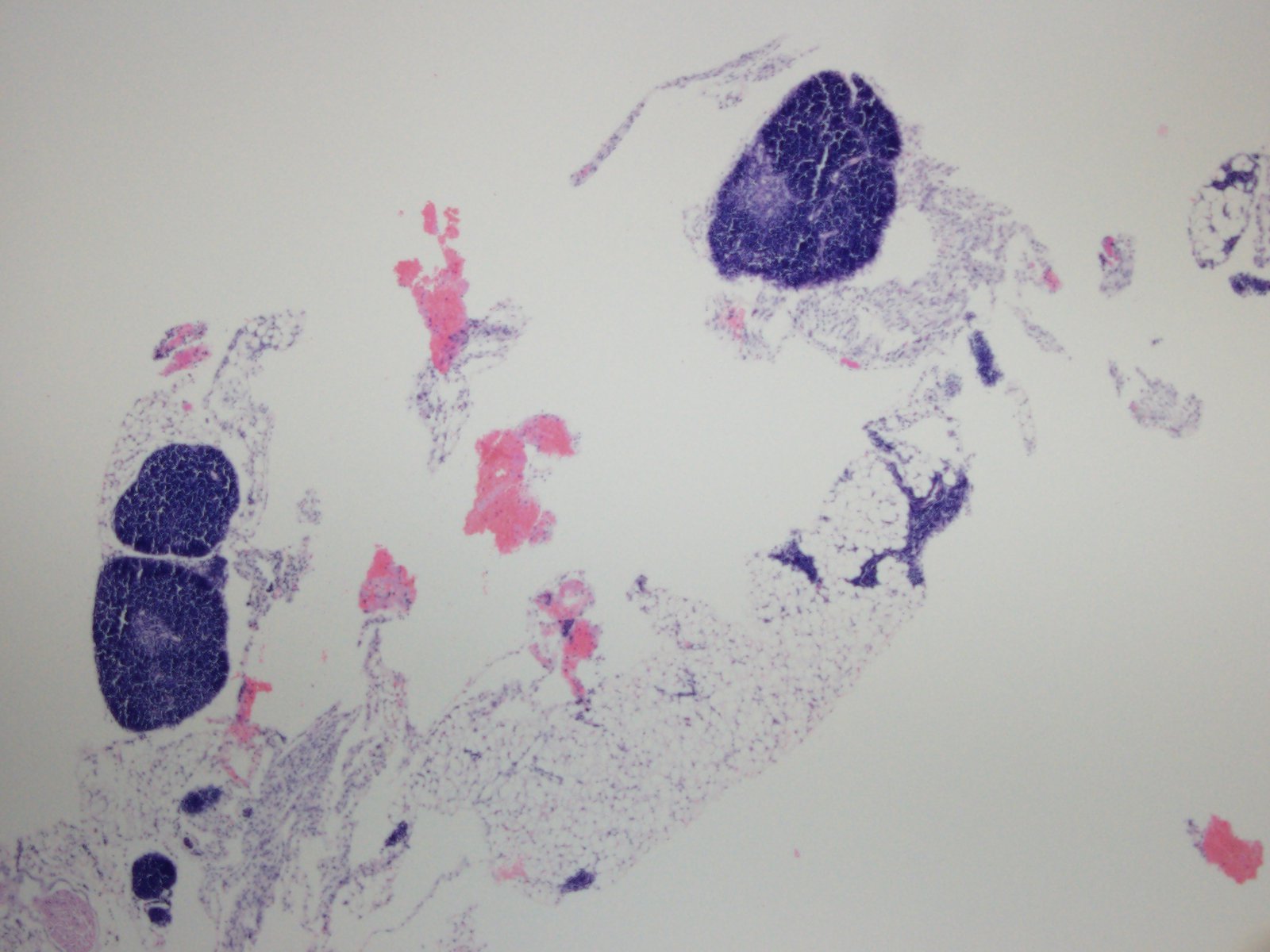  Myocardial fibers without microscopic changes. Minimal perivascular inflammatory infiltrates of the chronic type in the myocardium. Inflammatory infiltrates (0-1). | 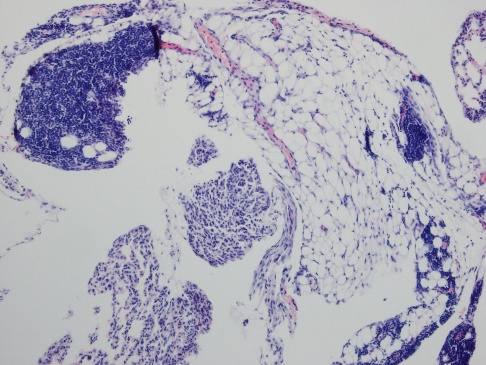  Alveoli and interstitial tissue without changes. Inflammatory infiltrates of the chronic type around pulmonary bronchioles and blood vessels.  Inflammatory infiltrates (2). | 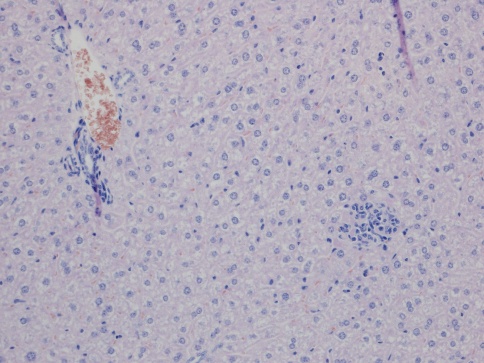  Hepatocytes without changes. Minimal inflammatory infiltrates of chronic type in portal-biliary spaces. Inflammatory infiltrates (0-1). | 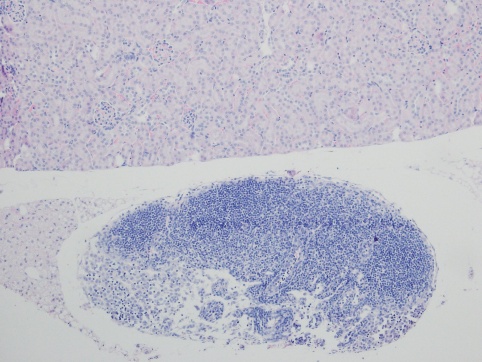  Glomeruli and renal tubules unchanged. Inflammatory infiltrates of chronic type (lymphocytes) in perinephric adipose tissue.  Inflammatory infiltrates (2). | 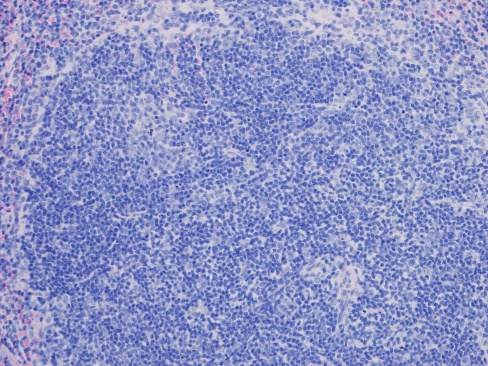  Hyperplasia of the white pulp of the spleen. Large lymphoid follicles  with germinal centers (2). |
| Group 7  EVs (Dose 3) | 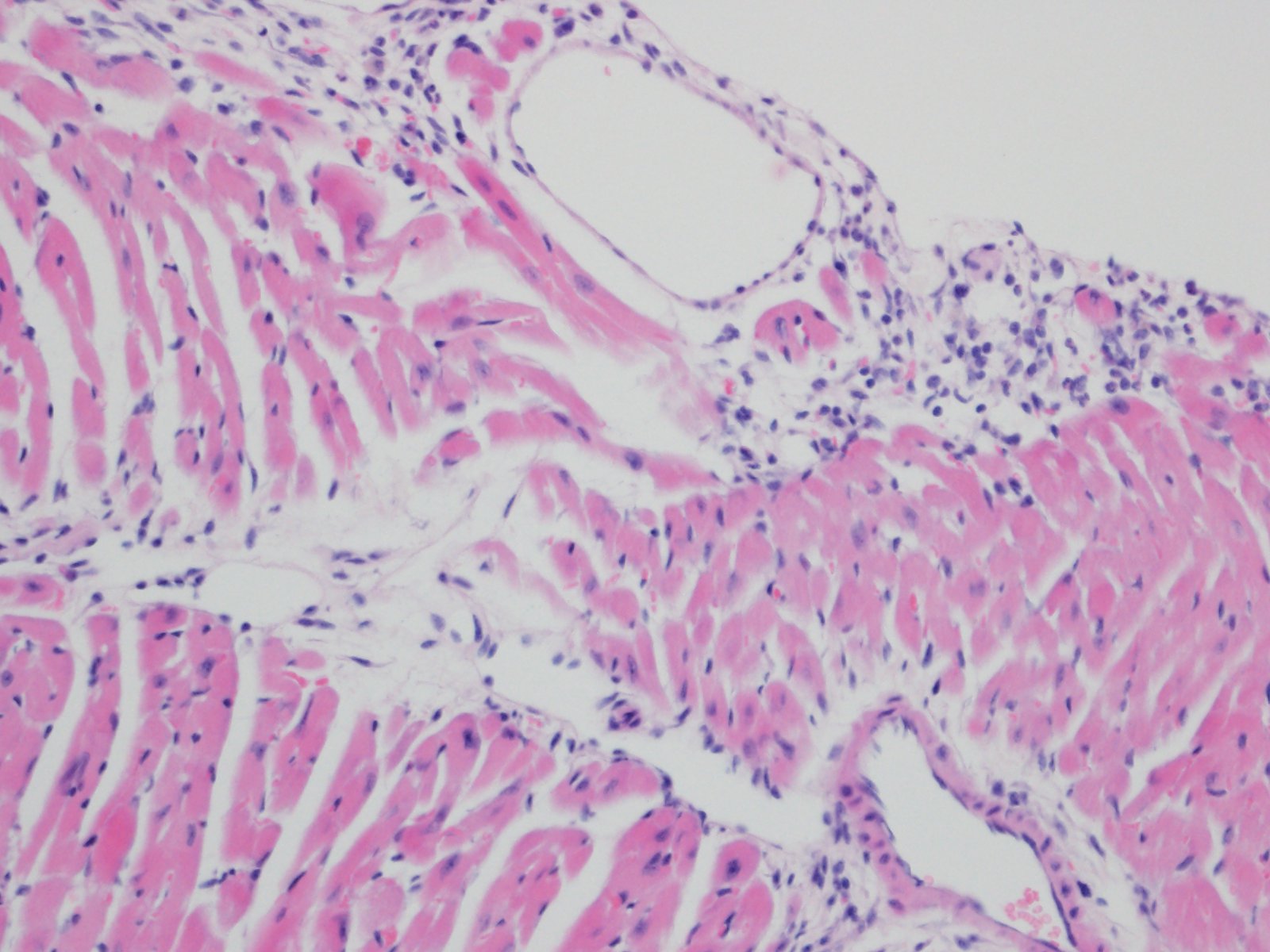  Myocardial fibers without microscopic changes. Inflammatory infiltrates of the chronic type of pericardial fatty tissue. Inflammatory infiltrates (2). | 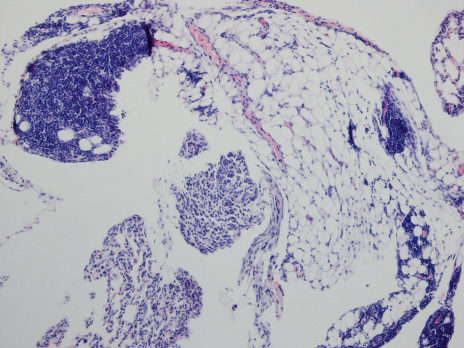  Alveoli and interstitial tissue without changes. Inflammatory infiltrates of the chronic type around pulmonary bronchioles and blood vessels. Inflammatory infiltrates (1). | 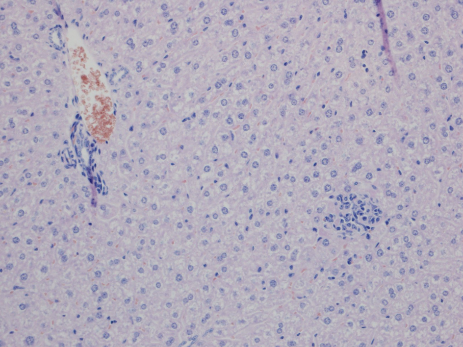  Hepatocytes without changes. Inflammatory infiltrates of chronic type in portal-biliary spaces.  Inflammatory infiltrates (1). | 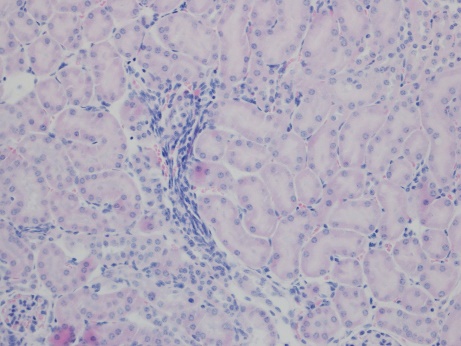  Glomeruli and renal tubules unchanged Minimal inflammatory infiltrates of the chronic type in the interstitial tissue of the kidney.  Inflammatory infiltrates (0-1). | 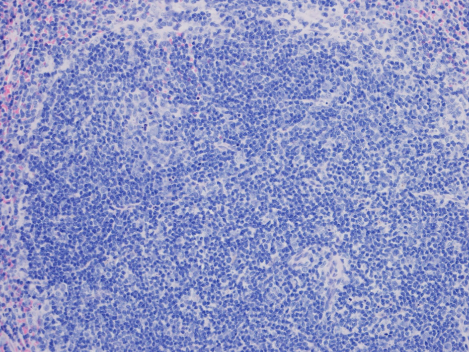  Hyperplasia of the white pulp of the spleen. Large lymphoid follicles with germinal centers (2). |
| Group 8  EVs (Dose 1)  +  KLH hemocyanin  (immunostimulation | 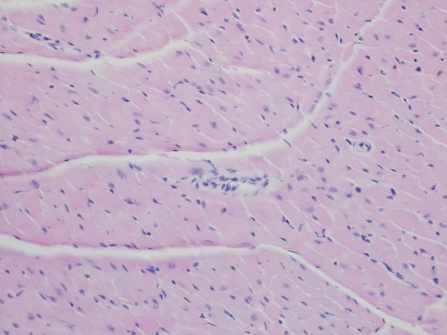  Myocardial fibers without microscopic changes. Minimal perivascular inflammatory infiltrates of the chronic type in the myocardium.  Inflammatory infiltrates (0-1). | 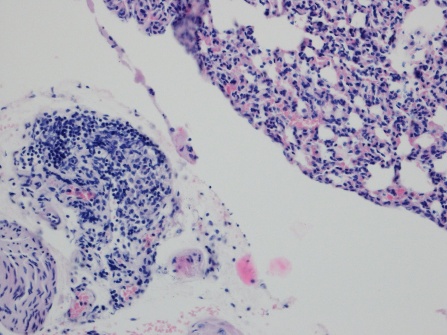  Alveoli and interstitial tissue without changes. Inflammatory infiltrates of the chronic type around pulmonary bronchioles and blood vessels.  Inflammatory infiltrates (1). | 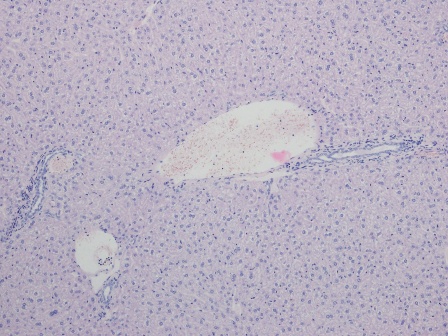  Hepatocytes without changes. Inflammatory infiltrates of chronic type in portal-biliary spaces.  Inflammatory infiltrates (1). | 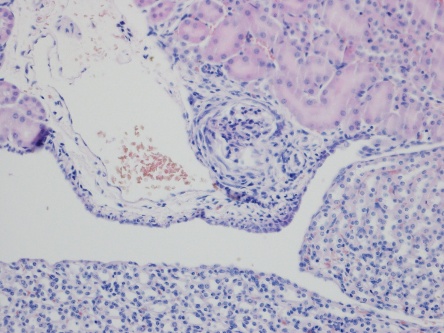  Glomeruli and renal tubules unchanged Minimal inflammatory infiltrates of the chronic type in the interstitial tissue of the kidney.  Inflammatory infiltrates (0-1). | 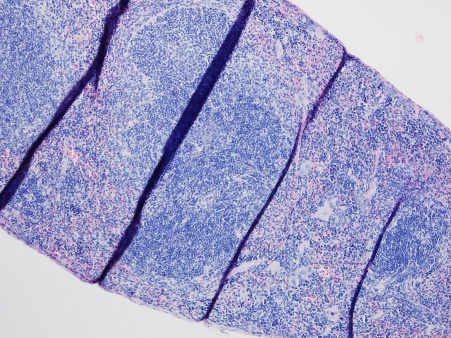  Hyperplasia of the white pulp of the spleen. Large lymphoid follicles ules with germinal centers (1). |
| Group 9  EVs (Dose 2)  +  KLH hemocyanin  (immunostimulation | 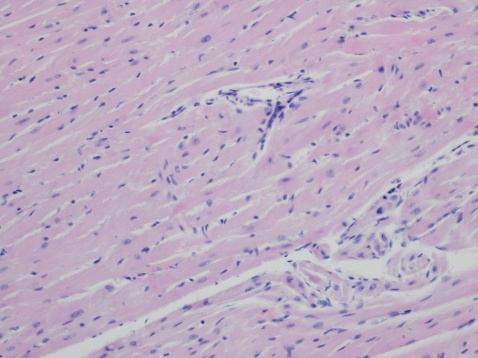  Myocardial fibers without microscopic changes. Minimal perivascular inflammatory infiltrates of the chronic type in the myocardium.  Inflammatory infiltrates (0-1). | 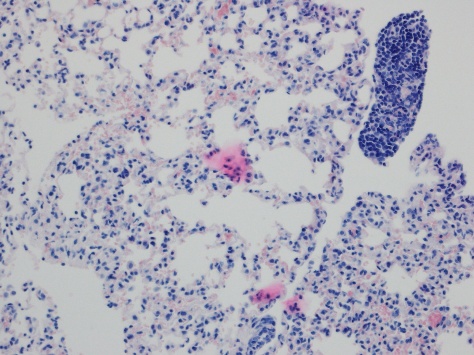  Alveoli and interstitial tissue without changes. Inflammatory infiltrates of the chronic type around pulmonary bronchioles and blood vessels.  Inflammatory infiltrates (1). | 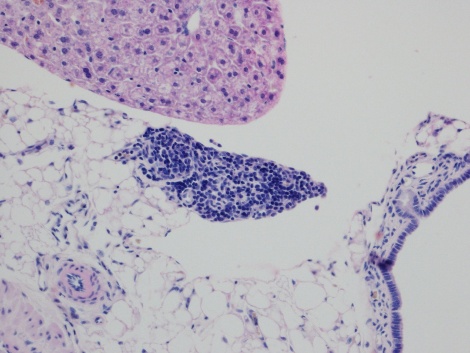  Hepatocytes without changes. Inflammatory infiltrates of the chronic type in portal-biliary spaces and in perihepatic adipose tissue  Inflammatory infiltrates (2). | 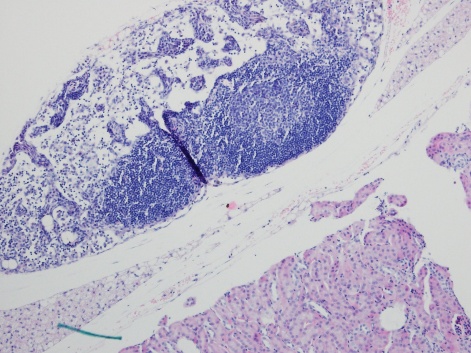  Glomeruli and renal tubules unchanged Inflammatory infiltrates of chronic type in perinephric fatty tissue.  Inflammatory infiltrates (2). | 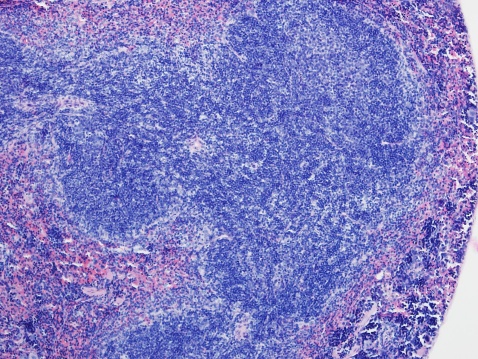  Hyperplasia of the white pulp of the spleen. Large lymphoid follicles with germinal centers (2). |
| Group 10  EVs (Dose 3)  +  KLH hemocyanin  (immunostimulation | 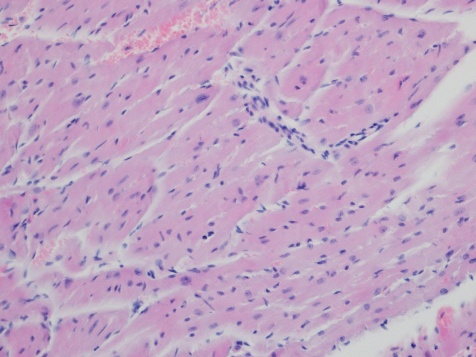  Myocardial fibers without microscopic changes. Minimal perivascular inflammatory infiltrates of the chronic type in the myocardium. Inflammatory infiltrates (0-1). | 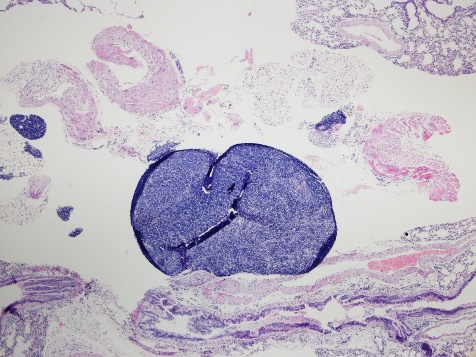  Alveoli and interstitial tissue without changes. Inflammatory infiltrates of the chronic type around the pulmonary bronchioles, blood vessels and in the fatty tissue of the pleura.  Inflammatory infiltrates (2). | 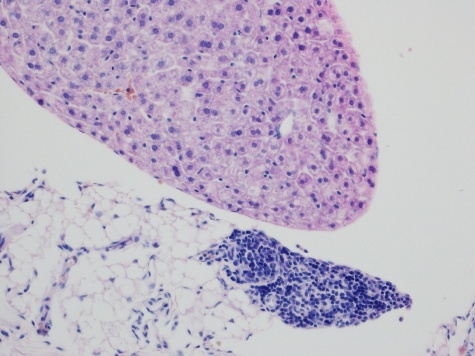  Hepatocytes without changes. Inflammatory infiltrates of the chronic type in portal-biliary spaces and in perihepatic adipose tissue  Inflammatory infiltrates (2). | 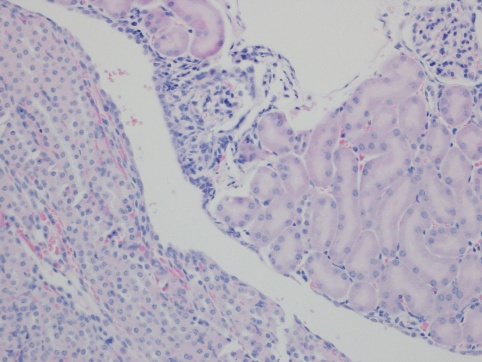  Glomeruli and renal tubules unchanged Minimal inflammatory infiltrates of the chronic type in the interstitial tissue of the kidney.  Inflammatory infiltrates (0-1). | 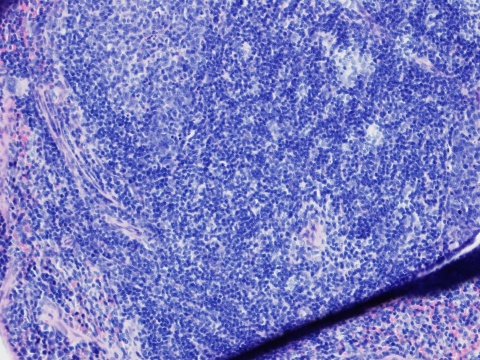  Hyperplasia of the white pulp of the spleen. Large lymphoid follicles with germinal centers (2). |
